# 9TH SRCA SYMPOSIUM ON THE CEREBELLUM: TAIPEI, MAY 2018

**DOI:** 10.1007/s12311-018-0953-2

**Published:** 2018-06-19

**PP1**

**Different types of multiple-synapse boutons in the cerebellar cortex between physically enriched and ataxic mutant mice**

Kim H. ^1^, Oh S.^1^, Lee S.^1^, Na J.^1^, Lee K.J.^2^, Rhyu I.J.^1^

^1^Department of Anatomy, Korea University College of Medicine, Seoul 02841, Korea ^2^ Laboratory of Synaptic Circuit Plasticity, Department of Structure & Function of Neural Network, Korea Brain Research Institute, Daegu 700-010, Korea

Corresponding author’s e-mail: irhyu@korea.ac.kr

Objectives: Experience-dependent synapse remodeling is associated with information storage in the nervous system. Neuronal synapses show alteration in various neurological and cognitive disorders in their structure and function. At the ultrastructural level, parallel fiber boutons contacting multiple spines of Purkinje cells in the cerebellar cortex are commonly observed in physiologically enriched animals as well as pathological ataxic mutants. However, the dendritic origin of those spines on parallel fiber multiple-synapse boutons has been poorly understood. Here we investigated this issue by 3-dimensional ultrastructural analysis to determine synaptic connectivity of multiple-synapse boutons in both mice housed in physically enriched environment and cerebellar ataxic mutants.

Materials and Methods: The MBSs from tottering mice and same background mice exposed in environmental enrichment were observed using serial sectioning transmission electron microscopy (ssTEM) and we analyzed dendritic spine origin. A three dimensional reconstruction image for the dendritic spine organization was performed.

Results and Conclusions: Our results demonstrated that environmental enrichment selectively induced multiple-synapse boutons to contact spines from the same parent dendrite, indicating focal strengthening of synapse through the simultaneous activation of two adjacent spines. In contrast, ataxic mutants displaying impaired motor coordination had significantly more multiple-synapse boutons involving spines originating from different neighboring dendrites compared to both wild-type and environmentally enriched animals, suggesting that compromising multiple synapse formation may lead to abnormal motor behavior in the mutant mice. These findings propose that environmental stimulation in normal animals mainly involves the refinement of preexisting synaptic networks, whereas pathological ataxic conditions may results from less-selective but compromising multiple synaptic formation. This study underscores that different types of multiple synapse boutons may have disparate effects on cerebellar synaptic transmission.

**PP2**

**The Yemenite Jewish Machado-Joseph Disease**

Gadoth N.^1, 3^, Gordon C. ^2, 3^, Zaltzman R. ^2^

^1^Departments of Neurology, Maynei Hayeshua Medical Center , Bnei Barak , Israel ^2^ Meir Medical Center, Kfar-Saba, Israel ^3^ The Sackler Faculty of Medicine, Tel-Aviv University, Israel

Corresponding author’s e-mail: gadoth@post.tau.ac.il

Objective: To describe the clinical and epidemiological features of the Jewish Yemenite pocket of Machado-Joseph disease in Israel

Background: Machado-Joseph Disease known also as SCA3 is an autosomal dominant progressive neurologic disorder due to a heterozygous (CAG)n trinucleotide repeat expansion encoding glutamine repeats in the ataxin-3 gene (ATXN3; on chromosome 14q32 , characterized principally by ataxia, spasticity, and ocular movement abnormalities (OMIM # 109150). Traditionally, all patients were considered as the offspring of two Portuguese families who lived in the Azorean islands i.e. the Machado and the Joseph families. Therefore, the disease was first known as Azorean Degeneration. In 1991, we have encountered a young man with progressive cerebellar ataxia, ophthalmoplegia, spastic qudriparesis, muscle atrophy and fasciculations who was a member of large Jewish Yemenite family which immigrated to Israel, from Taiiz in Yemen at the beginning of the 20th century. A meticulous clinical survey of the family disclosed a number of additional patients who previously received a variety of diagnoses, mainly cerebellar and or ponto-cerebellar atrophy. It took quite a while to come to the final conclusion reach to conclusion that this family of non-Portuguese origin is affected with SCA3. Later, when the mutation was mapped. the clinical diagnosis was genetically confirmed. Since then, we have diagnosed 65 patients from 17 different families and we are aware of additional 40 living patients in Israel who do not attend our clinic for personal reasons.

Conclusion: The Yemenite SCA3 which has a somewhat unique phenotype is the topic of our presentation

**PP3**

**Clinical visual characteristics in Wolfram syndrome**

An-Guor Wang ^1,2^, Hui-Chen Cheng ^1,2^, Ko-Ju Lin ^3^, Yann-Jinn Lee ^4^

^1^Department of Ophthalmology, Taipei Veterans General Hospital^2^Department of Ophthalmology, National Yang-Ming University School of Medicine^3^Department of Ophthalmology, Taipei Municipal Wanfang Hospital^4^Department of Pediatric, Mackay Memorial Hospital, and Mackay Medical College, Taipei, Taiwan

Corresponding author’s e-mail: agwang@vghtpe.gov.tw

Purpose: To characterize the clinical visual characterisitics in Wolfram syndrome

Methods: Retrospective review of the clinical records of Wolfram syndrome in the neuro-ophthalmic clinics of Taipei Veterans General Hospital from 2005 to 2017.

Results: A total of 5 cases were found with genetic confirmation of WFS1 gene mutations. Four males and one female patient were found, with an average age of 18.1 years. Case 1 and case 2 are siblings and have WFS1 gene mutations of c.1811G>A/c.2070_2079delCAGCCACCTG. Case 3 has gene mutations of c.683G>C/c.683G>C. Case 4 and Case 5 were siblings with mutations of c.1546_1548delTTC/c.2534_T>G. For those three patients who have been observed over 1 year, they have onset of optic nerve involvement at 9.3 years of age. Their visions had a mean logMAR of 0.43 at onset, which deteriorated to logMAR 1.11 within of 7.3 years.

Conclusions: Visual impairment typically onsets at 10 years of age in Wolfram syndrome. It commonly progressed gradually, and the patients will become legally blind in a few years.

**PP4**

**Study of CAG expansion in Spinocerebellar Ataxia 17 in Singapore**

Lisa Helen Ong ^1^, Xuan Ting Poh ^4^, Yong Qi Lee ^4^, Weiying Sim ^1^, Hai Yang Law ^2^, Eng King Tan ^3^, Yi Zhao ^1^

^1^Singapore General Hospital, Singapore^2^KK Women’s and Children’s Hospital, Singapore^3^National Neuroscience Institute, Singapore^4^School of Life Sciences and Chemical Technology, Ngee Ann Polytechnic 

Corresponding author’s e-mail: ong.lisa.helen@sgh.com.sg

Introduction and Objective: Spinocerebellar ataxia 17 (SCA17) is a condition with the expansion of CAG/CAA repeats in the coding region of the TATA box binding protein (TBP) leading to an abnormal expansion of polyglutamine in the corresponding protein. The diagnosis of SCA17 relies on molecular genetic testing to detect the abnormal CAG repeat expansion in TBP gene. In literature, affected individuals usually have more than 49 repeats and develop neurologic and psychiatric symptoms such as ataxia, dementia and involuntary movements, including chorea and dystonia. To date, limited information is available in Asians hence molecular genetic analyses of SCA are performed routinely in Singapore. Ataxia patient underwent SCAs (1, 2, 3, 6, 7, FA and DRPLA) genetic testing for nucleotide repeat expansion. Approximately 30% of the patient have been successfully identified and classified while the remaining 70% SCA suspected patient were further screened for other SCAs (8, 10, 12, 17, 36). SCA17 drew our interest as the pathologic repeat number as low as 27 overlap with the normal repeat numbers.

Methodology: A total of 524 subjects were involved in the analysis with 240 healthy individuals and 284 SCA suspected individuals. Up to 80% of the subjects are Chinese. Genetic analyses of CAG expansion frequency in SCA17 gene were performed on all samples. CAG repeat region of SCA17 were amplified with a pair of primers, the forward primer was labeled with fluorescent dye. The number of CAG repeats was detected by capillary electrophoresis followed by GeneScan analysis based on an internal size standard.

Results and Conclusion: Published data has shown that healthy individuals have CAG repeat range between 25 and 40 in SCA17 gene. The results of SCA17 screening showed that majority of the samples contain CAG repeats within the normal range in both groups. However, 9 SCA suspected individuals carried 40 to 44 CAG repeats and 13 healthy individuals were also presented with 40 to 47 CAG repeats. The results suggest that normal repeats range should be higher than 40 in local populations. The CAG repeats range may be variable in different populations. In conclusion, a higher definite cutoff value should be established for SCA17 patients due to higher repeat number in the healthy individuals.

**PP5**

**Analysis of PDE10A mutations in suspected Huntington Disease patients with normal CAG repeats in Singapore**

Weiying Sim ^1^, Lisa Helen Ong ^1^ , Hai Yang Law ^2^, Eng King Tan ^3^, Yi Zhao ^1^

^1^ Singapore General Hospital, Department of Clinical Translational Research, Singapore^2^ KK Woman’s and Children’s Hospital^3^ National Neuroscience Institute, Department of Neurology, Singapore

Corresponding author’s e-mail: sim.weiying@sgh.com.sg

Aim: Huntington disease (HD) is a rare neurodegenerative disorder, characterized by choreatic movements. It is caused by CAG repeat expansion in the huntingtin (HTT) gene. The clinical diagnosis of HD is based on clinical presentation, family history and genetic testing of the expansion with > 36 CAG repeats in HTT gene. Unfortunately, genetic testing for CAG expansion may not account for all HD patients. For example, there are patients who are clinically presented but do not carry pathogenic CAG range in HTT gene. Recently, there were evidences suggesting that PDE10A mutation was associated with HD. Comparing to healthy individuals, HD patients had reduced expression of PDE10A. As such, we aim to determine whether PDE10A could serve as a marker for our local suspected HD patients with normal CAG repeats in HTT gene.

Methodology: A total of 200 patients were suspected with HD and underwent genetic testing for CAG repeats in HTT gene. Out of 200 patients, 87 had CAG expansion in HTT gene and had been confirmed as HD positive. We selected 20 recently suspected HD patient for this study. The age of the patients ranged between 24 and 80 years old. 55% of them were Chinese and 60% were female. Next, we performed DNA sequencing on the reported hotspot areas of PDE10A gene, including exon 3, 4, 11 and 12.

Results and conclusion: Our study revealed that no mutation in PDE10A was found in the 20 subject samples, indicating that PDE10A might not be a good marker in Singapore. The result obtained was inconsistent with other reported studies. It could be due to low sample size in our study. The other possible reason could be HD is associated with different geographical regions. In conclusion, our study did not have the same finding as other reported studies that had demonstrated the association between PDE10A mutation and HD. As a result, we had yet to determine the diagnosis of those HD suspected patients as they neither carry pathogenic CAG repeats nor mutations in PDE10A.

**PP6**

**Comparable Progression of Spinocerebellar ataxias between Caucasians and Chinese: a reappraisal**

Yi-Cheng Lin, MD ^1^, Yi-Chung Lee, MD, PhD,^1,2,3^, Ting-Yi Hsu, MD, ^1^, Yi-Chu Liao, MD, PhD, ^1,2^* and Bing-Wen Soong, MD, PhD, ^1,2,3^*

^1^ Department of Neurology, Taipei Veterans General Hospital, Taipei, Taiwan^2^ Department of Neurology^3^Brain Research Center, National Yang-Ming University School of Medicine, Taipei, Taiwan

* These authors contributed equally to the manuscript

Corresponding author’s e-mail: bwsoong@ym.edu.tw

Objective: The aim of this study is reappraising the progression of five most common spinocerebellar ataxias (SCAs) among the Chinese and making a much-needed critical comparison with that of Caucasians.

Background: There have been very few longitudinal cohort studies of SCAs in Asians. The result of one earlier study by us demonstrating a faster progression of SCA in the Chinese than that of the Caucasians was intriguing.

Methods: Patients with SCA1, SCA2, SCA3, SCA6 or SCA17 were consecutively assessed using the scale for the assessment and rating of ataxia (SARA) for 5 years. The annual progression rates in SARA were compared among patients with different SCA subtypes by a linear mixed model and then compared in different populations. Predictors for the progression rates were analyzed.

Results: A total of 199 patients with SCA (10 SCA1, 37 SCA2, 118 SCA3, 25 SCA6 and 9 SCA17) were enrolled. The mean annual increase of SARA scores were 1.23 points in SCA1, 1.52 points in SCA2, 1.60 points in SCA3, 0.99 points in SCA6 and 3.26 points in SCA17. A larger CAG repeat length (>= 74) is associated with a faster progression in SCA3, whereas a lower total SARA score at first visit (<12) is associated with a faster clinical course in SCA6.

Conclusions: This study affirms that the annual progression rates of SCA2 and SCA3 are comparable between Caucasians and Han-Chinese. SCA17 has a progression rate twice as fast as that of SCA3.

**PP7**

**Modifying genes of SCA patients in mainland China**

Hong Jiang. ^1^, Zhao Chen^ 1^, Beishang, Tang^.1^

^1^Department of Neurology Xiangya Hospital, Central South University, Changsha, Hunan, 410008, P. R. China

Corresponding author’s e-mail: jianghong73868@126.com

Objective: Machado-Joseph disease (MJD), is a polyglutamine (polyQ) disease caused by a (CAG)n expansion in the ATXN3 gene.Only 50-70% of the variation in AO is explained by the CAG repeat expansion, suggesting that environmental and genetic factors may contribute to its phenotypic variability.

Materials and Methods: A total of 802 patients with MJD were enrolled in our study. Age at onset was defined by appearance of the first motor symptoms.All MJD patients were genotyped for ATXN3 and nine other polyQrelated genes (ATXN1, ATXN2, CACNA1A, ATXN7, TBP, ATN1, KCNN3, RAI1 and HTT).Analysis of the CAG repeats related to the AO of SCA3/MJD were performed using ANCOVA and multiple regressive. The overall association with age at onset for these modules was estimated by combining the association P-values for each allele.

Result: The longer allele of ATXN3 contributed to 56.9% variation of AO for SCA3/MJD. The shorter allele of ATXN3 with CAG repeats <19 or ranging from 26 to 40, contributed to 1.3% and 1.9% variation of AO respectively. The longer allele of ATXN3 interacting with the shorter allele of ATXN1, the difference of IT15 alleles and the difference of KCNN3 alleles contributed to 0.4%, 0.7% and 0.8% variation of AO respectively. Subjects with an intermediate ATXN2 allele ranging from 27 to 32, which contributed to 23.2% variation of AO, had an earlier AO (about 2.48 ± 1.58 years). The intermediate CACNA1A homozygous alleles ranging from 9 to 17 in SCA3/MJD patients contributed to 4.8% variation of AO. The shorter allele of ATXN7 with CAG repeats <10 or the longer allele ranging from 10 to 15 in SCA3/MJD patients contributed to 2.5% and 1.6% variation of AO respectively. The longer allele of ATXN7 with CAG repeats ranging from 12 to 17 and the shorter allele (>10) in SCA3/MJD individuals contributed to 3.8% variation. The shorter RAI1 allele with CAG repeats 12 or the longer allele ranging from 13 to 14 contributed to 0.7% and 5.2% variation of AO respectively. The longer allele of RAI1 with CAG repeats ranging from 13 to 14 and the shorter allele (>11) in SCA3/MJD individuals contributed to 7.3% variation. The network analysis shows ubiquitin C (UBC)-related genetic modules contributed to variation of AO in Chinese MJD patients

Conclusion: In Chinese Han population the CAG repeats in the longer allele of ATXN3 contribute to 56.9% variation of AO in SCA3/MJD. ATXN1, ATXN2, KCNN3, CACNA1A, ATXN7 and RAI1 gene may modulate the AO of SCA3/MJD. The genetic-network analysis offered a new perspective for the identification of pathway-based modifiers

**PP8**

**Updated frequency analysis of spinocerebellar ataxia in China**

Hong Jiang.^1^, Zhao Chen.^1^, Beisha Tang. ^1^

^1^Department of Neurology, Xiangya Hospital, Central South University, Changsha, Hunan, P. R. China

Corresponding author’s e-mail: jianghong73868@126.com

Objectives: To analyze updated frequency analysis of spinocerebellar ataxia in China.

Material and Method(s): A cohort of 1489 index ataxia cases in Chinese Han cohort (including 1009 dominant cases and 480 sporadic cases) were enrolled in our study.A list of genes implicated in SCAs were selected, including those carrying: (i) (CAG) n expansions: ATXN1 (SCA1), ATN1 (dentatorubral-pallidoluysian atrophy, DRPLA), ATXN2 (SCA2), ATXN3 (Machado-Joseph disease, MJD/SCA3), CACNA1A (SCA6), ATXN7 (SCA7), PPP2R2B (SCA12) and TBP (SCA17); (ii) other oligonucleotide expansions: ATXN8OS (SCA8), ATXN10 (SCA10), BEAN (SCA31) and NOP56 (SCA36); and (iii) conventional mutations: SPTBN2 (SCA5), CACNA1A, TTBK2 (SCA11), KCNC3 (SCA13), PRKCG (SCA14), ITPR1 (SCA15/16), KCND3 (SCA19/22), TMEM240 (SCA21), PDYN (SCA23), EEF2 (SCA26), FGF14 (SCA27), AFG3L2 (SCA28), ELOVL4 (SCA34), TGM6 (SCA35), ELOVL5 (SCA38), CCDC88C (SCA40), TRPC3 (SCA41) and CACNA1G (SCA42).

Result: Among 1009 dominant ataxia (SCA) probands, MJD/SCA3 (n = 632,62.64%) remained the most common subtype, followed by SCA2 (n = 88, 8.72%), SCA1 (n = 73, 7.23%), SCA6 (n = 17, 1.68%) and SCA7 (n = 11, 1.09%), SCA17 (n = 3,0.29%), SCA12 (n = 1, 0.09%) and DRPLA (n = 1, 0.09%). Among isolated patients, 18.91% could also be attributed to CAG expanded repeats. (CTA)n /(CTG)n expansions in ATXN8OS of SCA8 (n = 6, 0.59%) and (GGCCTG) n expansions in NOP56 of SCA36 (n = 8, 0.79%) were detected in dominant cases, while SCA36 was diagnosed in one isolated patient.Pathogenic variants were found in PRKCG for SCA14 (n = 1, 0.09%) and TGM6 for SCA35 (n = 2,0.19%) in the dominant cases; and de novo mutations were found in TMEM240 of SCA21 and CCDC88C of SCA40 in the isolated cases. Only 16.42% of dominant cases and as much as 80.49% of the isolated ones remained genetically undiagnosed.

Conclusion: We report updated relative frequency of SCA subtypes in dominant and isolated cases, in the largest sample described so far in China.The overall diagnostic yield is much higher in familial cases, though genes implicated in recessive ataxia may still need to be studied in the group of isolated patients.

**PP9**

**Two novel mutations implicate ITM2B as a new candidate gene underlying autosomal dominant cerebellar ataxia**

Ting-Yi Hsu ^1,2^, Yi-Chu Liao ^1,2^, Yi-Chung Lee ^1,2,3^, Yo-Tsen Liu ^1,2^, Bing-Wen Soong ^1,2,3,4^

^1^ Department of Neurology, Neurological Institute, Taipei Veterans General Hospital, Taipei, Taiwan^2^ Department of Neurology, National Yang-Ming University School of Medicine, Taipei, Taiwan^3^ Brain Research Center, National Yang-Ming University^4^ Institute of Neuroscience, National Yang-Ming University

Corresponding author’s e-mail: laurel19601085@yahoo.com.tw

Background: The ITM2B gene encodes BRI2, a transmembrane protein which can inhibit beta-amyloid deposition. ITM2B mutations have been identified to be associated with familial British dementia and familial Danish dementia, autosomal dominant diseases characterized by progressive dementia, cataracts and other ocular disorders. ITM2B may play important roles in cerebellar function because mutated BRI2 deposits extensively in the Purkinje cells of the cerebellum in patients of familial dementia and the BRI2 expression was reduced in patients with multiple sclerosis involving the cerebellum. Still, it is not clear if ITM2B mutations can cause hereditary cerebellar ataxias.

Objective: This study aimed to investigate the prevalence of ITM2B mutations in a cohort of Taiwanese patients with autosomal dominant cerebellar ataxias (ADCA).

Methods: Ninety-three individuals, selected from 199 ADCA patients who had been excluded for SCA1, 2, 3, 6, 7, 8, 10, 12, 17, 19/22, 31, 35, 36 and dentatorubral pallidoluysian atrophy (DRPLA), were recruited for ITM2B mutation analysis by targeted sequencing covering the coding exons and flanking introns of the gene.

Results: Two novel heterozygous ITM2B mutations were identified in three unrelated families. Two probands had the c.598C>G;p.His200Asp mutation while one family carried the c.800G>T;p.Ter267LeuextTer11 mutation. All patients manifested cerebellar ataxia. There was no evidence of dementia at least after being followed up to 48~68 years of age. Our study broadened the phenotypic spectrum of ITM2B mutations and implicated ITM2B as a new candidate gene underlying ADCA.

**PP10**

**Frequency of SYNE1 ataxia and extending the mutational spectrum in Korean population**

Ji Sun Kim, MD, PhD ^1,2^, Jinyoung Youn, MD ^1,2^, Jin Whan Cho, MD, PhD ^1,2*^

^1^ Department of Neurology, Samsung Medical Center, Sungkyunkwan University School of Medicine, Seoul, Korea^2^ Neuroscience Center, Samsung Medical Center, Seoul, Korea

Corresponding author’s e-mail: sunnyfor@hanmail.net

Introduction: There are great differences in the prevalence of the different forms of ataxias in different geographic regions, so the diagnosis should be considered based on ethnicity and region. Although autosomal recessive ataxias are slightly more frequent, with an estimated prevalence of 5 in 100,000, than autosomal dominant form worldwide, it is extremely rare in east Asia. Recent advance of new generation sequencing technique have been made in uncovering the new genetic causes and SYNE1 gene was identified as a cause of pure cerebellar ataxia. In this study, we aimed to explore the frequency of SYNE1 mutation in Korean population, and extend the mutational and phenotypic spectrum.

Methods: 63 Subjects were screened for SYNE1 mutations by new generation sequencing.

Results: We identified four novel mutations (1 splicing, 1 truncating and 2 missense mutation) spread throughout the SYNE1 gene from 2 patients (3.2%). The phenotype was mainly pure cerebellar ataxia in both cases. However, axonal neuropathy, mild frontal dysfunction and autonomic dysfunction were revealed. Age of onset was relatively late and disease course was rarely progressed.

Conclusion: Our results indicate SYNE1 mutations are not an uncommon cause of recessive ataxia with or without additional clinical features in Korean population.

**PP11**

**Molecular characterization of cerebellar ataxia associated KCND3 mutations**

HSIAO Cheng-Tsung. ^1,2,3*,^ FU Ssu-Ju. ^1^, LEE Yi-Chung. ^2,5,6^, TANG Chih-Yung. ^1,7^, JENG Chung-Jiuan. ^4,6,^ SOONG Bing-Wen.^ 2,5,6,8^

^1^ Graduate Institute of Physiology, College of Medicine, National Taiwan University^2^ Department of Neurology, National Yang-Ming University School of Medicine^3^ Division of Neurology, Department of Internal Medicine, Taipei Veterans General Hospital Taoyuan Branch^4^ Institute of Anatomy and Cell Biology, National Yang-Ming University School of Medicine^5^ Department of Neurology, Taipei Veterans General Hospital^6^ Brain Research Center, National Yang-Ming University^7^ Graduate Institute of Brain and Mind Sciences, College of Medicine, National Taiwan University^8^ Institute of Neuroscience, National Yang-Ming University

Corresponding author’s e-mail: hsiaoct26@gmail.com

Objectives: Voltage-gated potassium channel subfamily D member 3 (KV4.3) is encoded by human KCND3 gene. The aim of this study is to investigate the molecular characteristics of cerebellar ataxia associated KCND3 mutations.

Material and Methods: Plasmid for WT and mutant KV4.3 as well as KV channel-interacting protein 2 (KChIP2) were prepared. cRNAs of KV4.3 and KChIP2 were injected into Xenopus oocytes for electrophysiological studies utilizing two-electrode voltage clamp. HEK 293T cells expressing the KV4.3 and KChIP2 were analyzed by western-blotting, cycloheximide chase assays and biotinylation studies to understand the protein expression, degradation and membrane trafficking. Immunofluorescence for analyzing the subcellular distribution of the proteins was also performed.

Results: Mutant KV4.3 behaves as loss of function channels in the presence or absence of KChIP2. KV4.3 mutations display dominant-negative effects on wild type protein, result in not only significantly reduced functional expression but also altering voltage-dependent gating. The window currents of WT/mutant heterotetramers were significantly reduced, indicating the physiologically available channels were decreased in the presence of mutant KV4.3. With western-blotting analyses and cycloheximide chase assays, mutant KV4.3 demonstrate disrupted expression and rapider degradation. KV4.3 mutations also exert dominant-negative effects on protein expression, degradation and membrane trafficking of WT KV4.3. These conditions were not able to be rescued by KChIP2. Immunofluroscence images demonstrate insufficient membrane expression of the mutant KV4.3 protein as well as KChIP2.

Conclusion: We verified the functional alteration of cerebellar ataxia associated KCND3 mutations (C317Y, V338E, P375S and T377M). We concluded that the changes in electrophysiological properties as well as disrupted protein biosynthesis were underlying the pathogenesis of KCND3 mutation related cerebellar ataxia.

**PP12**

**Results of preliminary discussions of the Task Force on the Classification of Cerebellar Ataxias**

Beaudin M. ^1^, Luiz-Pedroso J. ^2^, Matilla-Duenas A. ^3^, Soong B.W. ^4^, Mitoma H. ^5^, Tsuji S. ^6^, Schmahmann J.D. ^7^, Manto M. ^8^, Rouleau G.A. ^9^, Klein C. ^10^, Dupre N. ^1^

^1^ Department of Neurological Sciences, CHU de Québec – Université Laval, Québec, QC, Canada ^2^ Department of Neurology, Federal University of Sao Paulo, Sao Paulo, SP, Brazil ^3^ Department of Neurosciences, Health Sciences Research Institute Germans Trias i Pujol (IGTP) – Universitat autonoma de Barcelona, Barcelona, Spain ^4^ Department of Neurology, National Yang-Ming University School of Medicine, Taipei, Taiwan, Republic of China ^5^ Tokyo Medical University, Medical Education Promotion Center, Tokyo, Japan ^6^ Department of Molecular Neurology, University of Tokyo, Tokyo, Japan ^7^ Department of Neurology, Massachusetts General Hospital and Harvard Medical School, Boston, MA, USA ^8^ Unité d’Etude du Mouvement (UEM), FNRS, ULB-Erasme, Bruxelles, Belgium ^9^ Department of Neurology and Neurosurgery, McGill University, Montreal, Qc, Canada ^10^ Department of Neurology, Mayo Clinic, Rochester, MN, USA

Corresponding author’s e-mail: marie.beaudin.1@ulaval.ca

Objective: The classification of hereditary recessive ataxias represents a challenge due to the large number of neurological and metabolic diseases that present with cerebellar dysfunction and to the phenotypic heterogeneity in known genetically defined disorders. Nevertheless, proper classification of genetic disorders is required to revise nomenclature, enlighten next generation sequencing panel design, and properly define this research field.

Material and Method: We recently published a systematic review of autosomal recessive disorders presenting with ataxia in order to propose a basis for classification of this group of disorders. We regrouped a panel of 11 international ataxia experts to create a Task Force on the Classification of Cerebellar Ataxias endorsed by the Society for Research on the Cerebellum and Ataxias. Up to now, Task Force members have shared ideas on general orientations, specific diseases, and nomenclature issues.

Results: Based on these discussions, we present an updated version of the classification of autosomal recessive ataxias. Disorders were classified into two groups, those that should be considered primary recessive ataxias and named accordingly, and those that belong to other groups of disorders but should be included in the differential diagnosis of a patient presenting with ataxia. The Task Force members will receive the updated list and discuss specific elements at the SRCA meeting to refine the classification and reach a consensus.

Conclusion: The objective of the Task Force is to create a consensus among experts in the field to propose a revised classification and nomenclature that is adapted to both clinical and research settings.

**PP13**

**Role of Meis1 in the granule cell development**

Tomoo Owa,^1^ Shinichiro Taya, ^1^ Satoshi Miyashita,^1,2^ Tomoki Nishioka, ^3^ Ryo Goitsuka, ^4^ Takuro Nakamura, ^5^ Kozo Kaibuchi, ^3^ and Mikio Hoshino ^1^

^1^ Department of Biochemistry and Cellular Biology, National Institute of Neuroscience, National Center of Neurology and Psychiatry, Japan

^2^ Department of Electrical Engineering and Bioscience, Waseda University, Japan^3^ Department of Cell Pharmacology, Nagoya University Graduate School of Medicine, Japan^4^ Division of Development and Aging, Research Institute for Biomedical Science, Japan^5^ Division of Carcinogenesis, Cancer Institute, Japanese Foundation for Cancer Research, Japan

Corresponding author’s e-mail: owa@ncnp.go.jp

Role of Meis1 in the granule cell development:

We report that myeloid ectopic viral integration site 1 homolog (Meis1) plays pivotal roles in the regulation of mouse granule cell (GC) development. Here, we show Meis1 is expressed in GC precursors (GCPs) and GCs during development. Our knock-down and conditional knock-out (cKO) experiments and in vitro assays revealed that Meis1 is required for proper cerebellar structure formation and for Pax6 transcription in GCPs and GCs. The Meis1–Pax6 cascade regulates the morphology of GCs. In the cKO cerebella, Smad proteins and bone morphogenic protein (BMP) signaling are severely reduced and Atoh1-expressing GCPs are ectopically detected in the inner external granule layer. These findings suggest that Meis1 regulates degradation of Atoh1 viaBMP signaling, contributing to GC differentiation in the inner EGL, and should provide understanding into GC development.

**PP14**

**All phases of climbing-fiber-Purkinje-cell synapse elimination require granule cells.**

Bailly Y. ^1^, Rabacchi S. ^2^, Sherrard R. M. ^2 3^, Rodeau J.-L. ^1^, Gasman S. ^1^, Vitale N. ^1^, Lohof A. M. ^2^, Mariani J. ^2 3^

^1^ INCI, CNRS UPR3212, Université de Strasbourg, Strasbourg, France ^2^ Sorbonne Universités UPMC Paris 06 and CNRS, IBPS, UMR 8256, Biological Adaptation and Ageing, Paris, France ^3^ APHP, DHU FAST, Institut de la longévité, 94205, Ivry-Sur-Seine, France

Corresponding author’s e-mail: byan@inci-cnrs.unistra.fr

Different afferent synapse populations interact to control the specificity of connections during neuronal circuit maturation. The elimination of all but one climbing-fiber synapse onto each Purkinje cell during the development of the cerebellar cortex is a particularly well studied example of synaptic refinement. The suppression of granule cell precursors by X irradiation during postnatal days 4 to 7 prevents this synaptic refinement, indicating a critical role for granule cells. Many studies of cerebellar development have suggested that synapse elimination has two phases, only one of which requires granule cells. In this study, we show that restricted irradiation on postnatal days 5 or 6 completely abolishes synaptic refinement, leaving some Purkinje cells in the adult with up to 5 climbing fibers. This indicates that the entire process of climbing fiber synapse elimination requires the presence of granule cells. The specific critical period for this effect appears to be directly related to regional timing differences of Purkinje cell and granule cell development in different cerebellar lobules, indicating a close, spatiotemporal synchrony between granule-cell development and olivo-cerebellar synaptic maturation.

**PP15**

**meis1 coordinates cerebellar granule cell development**

Owa T^1^, Taya S^1^, Miyashita S^1^, Nishioka T^2^, Goitsuka R^3^, Nakamura T^4^, Kaibuchi K^2^, Hoshino M^1^

^1^ Department of Biochemistry and Cellular Biology, National Institute of Neuroscience, National Center of Neurology and Psychiatry, Tokyo 187-8502, Japan

^2^ Department of Cell Pharmacology, Nagoya University Graduate School of Medicine, Showa-ku, Nagoya 466-8550, Japan ^3^ Division of Development and Aging, Research Institute for Biomedical Science, Tokyo University of Science, Noda, Chiba, Japan ^4^ Division of Carcinogenesis, Cancer Institute, Japanese Foundation for Cancer Research, Koto, Tokyo, Japan

Corresponding author’s e-mail: owa@ncnp.go.jp

Cerebellar granule cell precursors (GCPs) and granule cells (GCs) represent good models to study neuronal development. Here we report that the transcription factor, Meis1, plays multiple roles to regulate mouse GC development. We found that Meis1 is expressed in granule cell lineage cells and astrocytes in the cerebellum during development. Targeted disruption of the Meis1 gene specifically in the GC lineage resulted in smaller cerebella with disorganized lobules. Knockdown/knockout experiments for Meis1 as well as in vitro assays show that Meis1 binds to an upstream sequence of Pax6 to enhance its transcription in GCPs/GCs and further suggested that the Meis1-Pax6 cascade regulates morphology of GCPs/GCs during development. In the conditional knockout (cKO) cerebella, many Atoh1-positive GCPs were ectopically observed in the inner EGL, while similar phenomenon was also observed in cultured cerebellar slices treated with a BMP inhibitor. Furthermore, expression of Smad proteins as well as Smad phosphorylation were severely reduced in the cKO cerebella and Meis1-knockdown GCPs/cerebellar slices. Reduction of phosphorylated Smad was also observed in cerebellar slices electroporated with a Pax6 knockdown vector. Because it is known that BMP signaling induces Atoh1 degradation in GCPs, these findings suggest that the Meis1-Pax6 pathway increases the expression of Smad proteins to upregulate BMP signaling, leading to degradation of Atoh1 in the inner EGL, which contributes to differentiation from GCPs to GCs. Thus, this work reveals multiple functions of Meis1 in GC development and gives insights into the general understanding of the molecular machinery underlying neural differentiation from neural progenitors.

**PP16**

**The role of autism susceptibility candidate 2 (Auts2) in the cerebellar Purkinje cell development**

Kunihiko Yamashiro. ^1, 2^, Ryo Aoki. ^1, 2^, Asami Sakamoto. ^1^, Kei Hori. ^1^, and Mikio Hoshino. ^1, 2^

^1^ Department of Biochemistry and Cellular Biology, National Center of Neurology and Psychiatry (NCNP), Japan ^2^ Department of NCNP Brain Physiology and Pathology, Tokyo Medical and Dental University, Japan

Corresponding author’s e-mail: hoshino@ncnp.go.jp

Cerebellum has not only been a good model system to study the neural development, but also considered recently as one of the remarkable brain regions involved in higher brain functions such as the cognitions and sociability as well as various motor activities. Defects of cerebellar development have been associated with a variety of psychiatric disorders, but the underlying pathogenesis remains poorly understood.

Autism susceptibility candidate 2 (AUTS2) has been implicated in a variety type of psychiatric disorders such as autism, intellectual disabilities and schizophrenia. In developing CNS, Auts2 is highly expressed at several brain regions such as frontal cortex, hippocampus and cerebellum. Previously studies have demonstrated that AUTS2 is involved in the regulation of neuronal migration and neuritogenesis, acting as an upstream factor for Rac1 and Cdc42 during the late embryonic stages. In addition, the nuclear AUTS2 has been reported to participate in the regulation of gene expression by interacting with the Polycomb repressor complex 1 (PRC1) in the developing cerebral cortex. The physiological functions for AUTS2 in the postnatal brain development, however, remain largely unknown.

In this study, we investigated the role of AUTS2 in the postnatal cerebellar development. In the cerebellum, AUTS2 is specifically expressed in GABAergic neurons including Purkinje cells (PCs) and Golgi cells. The homozygotic mutant mice conditionally ablated Auts2 from the cerebellum exhibited the reduced size in the cerebellum, especially at the cerebellar hemispheres although the layer structure of cerebellar cortex appeared normal. In the Auts2 mutant cerebellum, the number of PCs was significantly decreased. Loss of Auts2 also resulted in the morphological abnormalities of PC dendrites as well as the defects of extensions and arborization. Immunohistochemical analysis revealed that the Auts2-deficient mice displayed the delay of the VGluT2-positive climbing fiber synapse formation at postnatal 2 weeks whereas the number of VGluT1-positive parallel fiber synapses was significantly increased. Moreover, Auts2 mutant mice exhibited the defects of several motor skills that included a mild locomotor ataxia, a hindlimb clasping upon tail suspension, and a decreased latency of fall off an elevated platform. These results suggest that AUTS2 plays a critical role for Purkinje cell development and maturation to acquire motor functions of the cerebellum.

**PP17**

**PDGF-C represents a new lineage marker for cerebellar stem cells**

Wu X., Liu W., Ding H.

Department of Biochemistry and Medical Genetics, University of Manitoba, Winnipeg, MB, Canada

Corresponding author’s e-mail: Hao.Ding@umanitoba.ca

PDGF-C is a newly identified ligand in the family of platelet-derived growth factor (PDGF). It has been demonstrated to signal through PDGF receptor-alpha important for mouse development. During mouse embryogenesis, PDGF-C has also been found to specifically express at the ventricular zone adjacent to the 4th ventricle which contains a group of cerebellar stem cells responsible for generating the majority of neurons and glia cells in the cerebellum. To demonstrate that PDGF-C-expressing cells could indeed function as cerebellar stem cells, in this study, we generated a Pdgf-cCreERT2 mouse strain, in which a tamoxifen-inducible Cre (CreERT2) cDNA was specifically targeted into the Pdgf-c genomic locus and controlled by the endogenous Pdgf-c regulatory elements. We also showed that Cre activity in this mouse strain could be specifically induced by tamoxifen, which allowed the fate of PDGF-C-expressing cells to be traced at various stages of cerebellar development. Using this lineage tracing tool, we demonstrated for the first time that PDGF-C-expressing cells located at the cerebellar ventricular zone could be multipoint, generating neuronal and glial progenitors that migrate radially into the cerebellum and give rise to most neurons, including Purkinje cells and deep cerebellar nuclei, and granule cells located in the internal granular layer. Our finding strongly suggest that PDGF-C could represent a new lineage marker for tracing and characterizing the developmental fate of cerebellar stem cells. Our study also implicates a role of PDGF signalling activity in the regulation of cerebellar stem cells.

**PP18**

**WDR4 deletion impairs cerebellar granule progenitor proliferation and causes ataxia**

Wu P.-R. ^1^, Gao W.-Z. ^1^, Cheng I-C. ^2^, Chou S.-J. ^2^, Chen R.-H. ^1^

^1^ Institute of Biological Chemistry, Academia Sinica, Taipei, Taiwan^2^ Institute of Cellular and Organismic Biology, Academia Sinica, Taipei, Taiwan

Corresponding author’s e-mail: f93448001@gate.sinica.edu.tw

Patients with mutations of WDR4, the noncatalytic subunit of a tRNA methyltransferase complex, have cerebellar atrophy and gait phenotypes. However, the underlying mechanisms remain unclear and the therapeutic strategy is limited. Using a mouse model, we report here that conditionally eliminating WDR4 in the central nervous system results in reductions of the size, the foliation, and the external granule layer of the cerebellum. Furthermore, the proliferation of cerebellar granule progenitors is decreased in the WDR4 loss-of-function mice whereas the apoptosis is not altered. Particularly, WDR4 deficiency blocks the S phase entry without affecting the cell cycle exit of the cerebellar granule progenitors. In addition, WDR4 loss-of-function mice display an ataxia phenotype before weaning. Together, our studies reveal a novel function of WDR4 on cerebellar neurogenesis, and thus provide a potential therapeutic strategy to treat the related neurodevelopmental disorders.

**PP19**

**Synapse elimination of all redundant climbing fibers requires granule cells in the postnatal cerebellum.**

Bailly Y. ^1^, Rabacchi S. ^2^, Sherrard R. M. ^2 3^, Rodeau J. L. ^4^, Demais V. ^1 5^, Vitale N. ^1^, Gasman S. ^1^, Lohof A. M. ^2^, Mariani J. ^2 3*^

^1^ Intracellular Membrane Trafficking in the Nervous and Neuroendocrine System, INCI, CNRS UPR3212, Université de Strasbourg, Strasbourg, France^2^ Sorbonne Université UPMC, CNRS, UMR 8256, Biological Adaptation and Ageing, B2A, 75005 Paris, France^3^ APHP, DHU FAST, Institut de la longévité, 94205, Ivry-Sur-Seine, France^4^ Nociceptive Signalling in the Spinal Cord, CNRS UPR3212, Université de Strasbourg, Strasbourg, France^5^ Plateforme d’Imagerie In Vitro, CNRS UPS 3156 Université de Strasbourg, Strasbourg, France

Corresponding author’s e-mail: byan@inci-cnrs.unistra.fr

Different afferent synapse populations interact to control the specificity of connections during neuronal circuit maturation. The elimination of all but one climbing-fiber synapse onto each Purkinje cell during the development of the cerebellar cortex is a particularly well studied example of synaptic refinement. The suppression of granule cell precursors by X irradiation during postnatal days 4 to 7 prevents this synaptic refinement, indicating a critical role for granule cells. Several studies of cerebellar development have suggested that synapse elimination has a first granule cell-independent phase and a second, granule cell-dependent phase. In this study, we show that restricted irradiation on postnatal days 5 or 6 completely abolishes synaptic refinement, leaving the olivo-cerebellar circuit in its immature configuration in the adult with up to 5 climbing fibers innervating the Purkinje cells in some cases. This implies that the putative early phase as well as the late phase of climbing fiber synapse elimination has been blocked by irradiation-induced granule cell loss and indicates that the entire process of climbing fiber synapse elimination requires the presence of granule cells. The specific critical period for this effect appears to be directly related to regional timing differences of Purkinje cell and granule cell development in different cerebellar lobules, indicating a close, spatiotemporal synchrony between granule-cell development and olivo-cerebellar synaptic maturation.

**PP20**

**RBM4 modulates radial migration via alternative splicing of Dab1 during cortex development**

Dhananjaya D, Kung-Yang Hung and Woan-Yuh Tarn.

Corresponding author’s e-mail: wtarn@ibms.sinica.edu.tw

The RNA-binding motif 4 (RBM4) protein participates in cell differentiation via its role in regulating the expression of or tissue-specific or developmentally regulated mRNA splice isoforms. RBM4 is expressed in embryonic brain during development; it is initially enriched in the ventricular zone/subventricular zone and subsequently distributed throughout the cortical cortex. Rbm4a knockout brain exhibited delayed migration of late-born neurons. Using in utero electroporation, we confirmed that knockdown of RBM4 impaired cortical neuronal migration. RNA immunoprecipitation-sequencing identified Disabled-1 (Dab1), which encodes a critical Reelin signaling adaptor, as a potential target of RBM4. Rbm4a knockout embryonic brain showed altered Dab1 isoform ratios. Overexpression of RBM4 promoted the inclusion of Dab1 exons 7 and 8 (7/8), whereas its antagonist PTBP1 acted in an opposite manner. RBM4 directly counteracted the effect of PTBP1 on exon 7/8 selection. Finally, we showed that the full-length Dab1, but not exon 7/8-truncated Dab1, rescued neuronal migration defects in RBM4-depleted neurons, indicating that RBM4 plays a role in neuronal migration via modulating the expression of Dab1 splice isoforms. Our findings imply that RBM4 is necessary during brain development and that its deficiency may lead to developmental brain abnormality.

**PP21**

**Using a detailed model to explore the importance of Purkinje cell dendrites for somatic firing properties**

Zang Y., De Schutter E.

Computational Neuroscience Unit, Okinawa Institute of Science and Technology Graduate University, Onna-son, Okinawa, Japan

Corresponding author’s e-mail: yunliang.zang@oist.jp

Purkinje cells (PCs), the sole output neurons of the cerebellum cortex, play a significant role in movement coordination, control and learning. Although PC electrophysiological properties are well explored, surprisingly the potential effect of dendrites on regulating somatic spikes has been largely ignored. Based on available experimental data, we have built a new PC model, which can reproduce a plethora of experimental observations. Using this model, we are able to reveal the underpinnings of experimentally observed phenomena and reconcile discrepancies between in vitro and in vivo observations.

We first demonstrated that dendrites can lower the metabolic cost of somatic spikes. We further found that dendritic calcium spikes evoked by climbing fiber (CF) input are modulated by the voltage states (or firing rates) of PCs, with a strong effect on somatic spikes. Seemingly paradoxically, concurrent inhibitory/excitatory synaptic inputs can increase/decrease the number of somatic spikelets by modulating the timing of dendritic spikelets.

Finally, we demonstrate the critical role of dendrites in determining the mysterious phase response curves (PRCs) observed in PCs. In concert with somatic ionic currents, dendrites regulate the somatic inter-spike interval membrane potentials at different firing rates, which give rise to the firing-frequency dependency of the PRC in PCs.

Therefore, our experimental data-based modeling results highlight the role of dendrites on somatic firing properties, which may be used to link the varied PC electrophysiological properties with abnormal development of dendritic trees under pathological conditions.

**PP22**

**Ocular motor function in stiff person syndrome - role of glutamic acid decarboxylase and beyond**

Wang H. ^1^, Nelson L. ^1^, Wang F. ^1^, Wilmot G.R. ^2^, Shaikh A.G. ^1^

^1^University Hospitals Cleveland Medical Center, Neurological Institute^2^ Emory University, Department of Neurology

Corresponding author’s e-mail: han.wang@uhhospitals.org

Objective: Delineate pathophysiology of gaze-holding function in stiff person syndrome (SPS).

Background: Glutamic acid decarboxylase (GAD) catalyzes conversion of glutamate to gamma-aminobutyric acid (GABA). Anti-GAD antibodies, as seen in SPS, can compromise this function. Proof of this mechanism comes from peculiar eye movements in SPS such as downbeat nystagmus, dysmetria, impaired pursuit/vestibulo-ocular reflex cancellation. It is possible that eye movement dysfunction in SPS is multifactorial, due to deficiency in GABA and excess of glutamate.

Methods: We measured gaze holding in 11 SPS patients using high-resolution oculography. Patterns of deficits were dissected via offline analysis of eye position. Specific waveforms and known principles of eye movement control systems were applied to determine the role of GABA versus glutamate.

Results: Downbeat nystagmus and horizontal gaze-evoked nystagmus were the most common deficits. Downbeat nystagmus presented in 9 patients, while 6 had horizontal gaze-evoked nystagmus. They were co-existent in some patients. Horizontal gaze-evoked nystagmus had velocity-decreasing waveform, and the slow-phase eye velocity reduced as the desired eye position reached central null position. Null position for vertical nystagmus varied in patients: in 2 it was downward, while in 7 it was upward. Seven patients with downbeat nystagmus had velocity-increasing waveform, and 2 had velocity-decreasing waveform. Two patients had opsoclonus. Visually guided saccades were slow in 2 patients with opsoclonus and 1 with normal gaze holding.

Conclusion: Deficiency in GABA causing dysfunction of brainstem neural integrator explains the eye-in-orbit position dependence of slow-phase velocity of horizontal and vertical nystagmus, null position and velocity-increasing/decreasing waveforms of downbeat nystagmus. GABA deficiency does not explain opsoclonus, which is putatively due to excessive glutamate causing reverberations of saccadic burst neurons

**PP23**

**Axonal spike bursting in the inferior olive is oscillatory state-dependent**

Tang A.D ^1^ and Uusisaari M.Y ^1^

^1^Okinawa Institute of Science and Technology Graduate University, Okinawa, Japan

Corresponding author’s e-mail: alexander-tang@oist.jp

Objectives: The inferior olive (IO) transmits information critical for motor timing to the cerebellar cortex via climbing fibres that initiate the characteristic complex spikes (CS) in Purkinje neurons (PNs). Information from the IO is transmitted as bursts whereby single somatic action potentials (APs) are followed by high frequency axonal spikes (“spikelets”). The number of spikelets in each IO spike burst is known to modulate the plasticity of the cerebellum, however, the factors that determine the number of spikelets are unclear. Furthermore, it is not known whether spikelet number modulation is similar in IO neurons in an oscillating and non-oscillating state. We investigated whether the intrinsic passive and active membrane properties of IO neurons determine the number of spikelets in neurons that are in an oscillating and non-oscillating state.

Materials and Methods: Whole-cell current-clamp recordings were made from mouse brainstem slices and spike analysis was restricted to cells displaying spontaneous APs (n = 29 cells).

Results: Preliminary analysis suggests that spikelet number in IO neurons that are in a non-oscillating state are negatively correlated to the AP threshold and the duration of the high–threshold calcium-related afterdepolarisation (ADP). IO neurons in an oscillating state had spikelets that were negatively correlated to the AP threshold but positively correlated to the ADP duration. In relation to the oscillation properties, IO spikes occurred mainly on the rising and falling phases and oscillations >10 mV had shorter ADPs and less spikelets.

Conclusions: Our results suggest that information transfer from the IO is in part dependent on the oscillatory state of the IO neuron with a potential functional threshold of oscillation amplitude, such that high amplitude oscillations result in small axonal bursts. This ongoing work helps to elucidate the function of IO oscillations and axonal spike bursting in cerebellar function and plasticity.

**PP24**

**Constitutive and activity-dependent AMPA-receptor trafficking in cerebellar Purkinje cell**

Kazuhiko Yamaguchi

Corresponding author’s e-mail: kazuhiko.yamaguchi@riken.jp

Exo/endocytosis and lateral diffusion of AMPA-type glutamate receptors (AMPARs) would regulate synaptic strength at the parallel fiber (PF)-Purkinje cell (PC) synapse in the cerebellum. Though some mathematical models　of synaptic plasticity in PC based on receptor-trafficking have been reported, however, realistic kinetic parameters of exo/endocytosis of AMPARs were not experimentally measured yet. Here, we measured a rate of elimination of AMPA-Rs from synaptic membrane of rat PC in slice preparation by uncaging of a caged inhibitory peptide, of which original form blocked exocytic insertion of GluA2-containing AMPAR into synaptic membrane. Photolysis of the caged-peptide, applied intracellularly through a whole-cell patch-pipette, by UV-irradiation caused rapid decrease in PF-EPSC amplitude, which was considered as elimination of AMPARs from the synaptic membrane. Elimination time-constant was around 1.3 min under basal condition. This elimination should be consisted of lateral diffusion and/or endocytosis of AMPARs. To estimate lateral diffusion component, uncaging was done during blockade of endocytosis by dynamin inhibitor. Surprisingly, no change was detected in PF-EPSC amplitude, suggesting that free lateral diffusion of AMPA-Rs from synaptic to extrasynaptic region was negligible. Next, we measured elimination rate during LTD. Elimination time-constant was not altered by LTD-inducing stimulation. On the other hand, exocytic insertion of AMPARs was markedly suppressed during LTD-expression. A model involving suppression of exocytosis of AMPARs can explain measured LTD quantitatively.

**PP25**

**Neuron-specific Sel1L deficiency induces a progressive cerebellar ataxia in mice**

Wensheng Lin

University of Minnesota

Corresponding author’s e-mail: linw@umn.edu

Endoplasmic reticulum (ER) is responsible for modification and folding of secretory proteins and membrane proteins. Protein folding in the ER is a delicate and error-prone process. Misfolded/unfolded proteins in the ER are recognized and degraded by a process known as ER-associated degradation (ERAD). The Suppressor/Enhancer of Lin-12-like (Sel1L) - hydroxymethylglutaryl reductase degradation protein 1(Hrd1) complex consisting of the E3 ubiquitin ligase Hrd1 and its adaptor protein Sel1L is highly conserved and the best-characterized ERAD machinery. Moreover, it has been demonstrated that Sel1L controls the stability of the Hrd1 protein and that Sel1L is essential and necessary for the ERAD activity of the Sel1L-Hrd1 complex. Interestingly, a recent report showed that a missense mutation of Sel1L leads to Purkinje neuron loss and progressive early-onset cerebellar ataxia in dogs. Herein, we generated a mouse model that allowed for temporally controlled deletion of Sel1L specifically in neurons, and found that neuron-specific Sel1L deficiency reduced the number of Purkinje neurons and the thickness of the molecular layer of the cerebellum and resulted in a progressive cerebellar ataxia. Thus, these data suggest the essential role of ERAD in maintaining Purkinje neuron viability and functions.

**PP26**

**Climbing Fiber-Purkinje Cell Synaptic Pathology Across Essential Tremor Subtypes**

Kuo S.H. ^1^, Lee D. ^1^, Gan S.R. ^1,2^, Pan M.K. ^3^, Faust P.L. ^4^, Louis E.D. ^5^

^1^ Columbia University, Department of Neurology, New York, United States^2^ Fujian Medical University, Department of Neurology, Fuzhou, China^3^ National Taiwan University, Department of Medical Research, TaipeiTaiwan^4^ Columbia University, Department of Pathology and Cell Biology, New York, United States^5^ Yale University, Department of Neurology, New Haven, United States

Corresponding author’s e-mail: sk3295@columbia.edu

Background: The cerebellum has been implicated in the pathophysiology of essential tremor (ET). ET is heterogeneous in nature and cases may be subdivided based on clinical features. ET patients may thus be subdivided by age of onset, family history of tremor, and presence of head tremor. We recently described climbing fiber-Purkinje cell (CF-PC) synaptic abnormalities in ET; however, these CF pathological features have not been studied across different ET subtypes.

Objectives: To explore whether these CF-PC synaptic abnormalities differ across ET subtypes.

Methods: We studied two climbing fiber (CF-PC) synaptic pathologies (CF synaptic density and percentage of CFs in the parallel fiber [PF] territory) in the cerebella of 60 ET cases with a range of clinical presentations and 30 age-matched controls.

Results: Compared to controls, ET cases had lower CF synaptic density and a higher percentage of CFs in the PF territory. ET cases with tremor onset < 50 years and tremor onset ≥ 50 years did not differ significantly with respect to CF synaptic density and percentage of CFs in the PF territory. Similar results were found when comparing familial vs. sporadic ET cases, and ET cases with head tremor vs. those without head tremor. Among all ET cases, lower CF synaptic density was associated with lower PC counts and higher torpedo counts. In addition, higher percentage of CFs in the PF territory was associated with lower PC counts and higher torpedo counts.

Conclusions: These findings support the notion that changes in the distribution of CF-PC synapses are broadly part of the neurodegenerative process in the ET cerebellum.

**PP27**

**Planar cell polarity gene Fuz triggers apoptosis in neurodegenerative diseases**

Chen Z.S. ^1^, Li L. ^1^, Peng S. ^1^, Chen F. ^1^, Zhang Q. ^1^, An Y. ^1^, Chan T.F. ^1^, Lau K.F. ^1^, Ngo J.C. ^1^, Wong W.T. ^1^, Kwan K.M. ^1,^ Chan H.Y.E. ^1^

^1^ School of Life Sciences, The Chinese University of Hong Kong, Hong Kong, China

Corresponding author’s e-mail: hyechan@cuhk.edu.hk

Objectives: Planar cell polarity (PCP) describes a cell-cell communication process through which individual cells coordinate and align on a tissue plane. We determined of the role of Fuz, a PCP gene, in neuronal apoptosis.

Results and conclusion : We found that endogenous Fuz was upregulated in patients with polyglutamine (polyQ) diseases. Disruption of endogenous Fuz function mitigated polyQ-induced neurodegeneration. We further discovered the transcriptional regulator Yin Yang 1 (YY1) associates with Fuz promoter. Overexpression of YY1 promoted Fuz promoter hypermethylation, causing transcriptional repression of Fuz. Importantly, soluble YY1 protein was diminished in levels in polyQ patients. Such reduction compromises the function of YY1, resulting in Fuz transcriptional derepression and induction of neuronal apoptosis. Taken together, this study unveils a generic Fuz-mediated apoptotic cell death pathway in neurodegenerative disorders.

**PP28**

**A quantitative study of “empty baskets” in essential tremor and cerebellar degenerative diseases**

Faust P.L ^1^, Lee J.S. ^2^, Louis E.D. ^2^

^1^ Columbia University Medical Center, Department of Pathology & Cell Biology, New York, USA ^2^ Yale University Medical School, Department of Neurology, New Haven, USA

Corresponding author’s e-mail: plf3@cumc.columbia.edu

Objective: To quantify empty basket pathology in the cerebellar cortex in essential tremor (ET), spinocerebellar ataxias (SCAs) 1, 2, 3, and 6 characterized by variable degrees of Purkinje cell (PC) loss, and healthy controls.

Background: The mechanisms that underlie ET are not completely understood. Recent postmortem studies have identified structural abnormalities in the ET cerebellum involving the PCs and their interneuron network. Normally, the axons of basket cells surround the PC soma, forming a basket plexus. In the setting of PC loss and relatively preserved basket cells, these baskets appear empty. Hence, “empty baskets” are a marker of PC loss

Design/Methods: Using a standard parasagittal, formalin-fixed tissue block from the neocerebellum, dual immunohistochemical staining for calbindinD28k and glutamic acid decarboxylase was performed on 102 brains (50 ET, 27 SCA and 25 controls). Quantitative microscopic analysis of empty basket pathology was performed blinded to diagnosis.

Results: The median percentage of empty baskets in ET cases was 1.5 times higher than that of age-matched controls (48.8% vs. 33.5%, p < 0.001). The median percentage of empty baskets was lower in ET compared to SCA1 (59.7%, p = 0.01), SCA2 (77.5%, p = 0.003), and SCA6 (87.0%, p < 0.001), indicating ET has an intermediate degree of empty basket cell pathology compared to diseases with extreme PC loss. PC loss is not a feature of SCA3, and the median percentage of empty baskets (30.1%) was similar to controls. As expected, the number of empty baskets per microscopic field was inversely related to PC counts (r = -0.793, p < 0.001).

Conclusions: Empty baskets are a disease-associated feature of ET. In comparison, several of the SCAs had even more empty baskets, although a spectrum existed across the SCAs. The empty basket pathology in ET was between that of controls and SCAs; hence, ET appears to be an intermediate state or less advanced state of cerebellar degeneration.

Study Supported by: National Institutes of Health R01 NS088257.

**PP29**

**The indole compound NC009-1 inhibits aggregation and promotes neurite outgrowth through enhancement of HSPB1 in spinocerebellar ataxia 17 cell models**

Lin C.H. ^a,1^, Chen W.L. ^a,1^, LIN T.H. ^b^, Chao C.Y. ^a,^ Chang K.H. ^a^, Chen C.M. ^a,*,^ Wu, Y.R. ^a,*,^ Lee-Chen G.J. ^b,*^

^a^ Department of Neurology, Chang Gung Memorial Hospital, Chang Gung University College of Medicine, Taipei 10507, Taiwan

^b^ Department of Life Science, National Taiwan Normal University, Taipei 11677, Taiwan; ^*^Corresponding author

^1^ Those authors contributed equally to this work. E-mail address: cmchen@adm.cgmh.org.tw (C.-M. Chen); yihruwu@adm.cgmh.org.tw (Y.-R. Wu); t43019@ntnu.edu.tw (G.-J. Lee-Chen)

Corresponding author’s e-mail: chli416@hotmail.com

Spinocerebellar ataxia type 17 (SCA17) is caused by the expansion of translated CAG repeat in the TATA box binding protein (TBP) gene encoding a long polyglutamine (polyQ) tract in the TBP protein, which leads to intracellular accumulation of aggregated TBP and cell death. The molecular chaperones act in preventing protein aggregation to ameliorate downstream harmful events. In this study, we used Tet-On cells with inducible SCA17 TBP/Q79-GFP expression to test five in-house NC009 indole compounds for neuroprotection. We found that both aggregation and polyQ-induced reactive oxygen species can be significantly prohibited by the tested NC009 compounds in Tet-On TBP/Q79 293 cells. Among the five indole compounds, NC009-1 up-regulated expression of heat shock protein family B (small) member 1 (HSPB1) chaperone to reduce polyQ aggregation and promote neurite outgrowth in neuronal differentiated TBP/Q79 SH-SY5Y cells. The increased HSPB1 thus ameliorated the increased BH3 interacting domain death agonist (BID), cytochrome c (CYCS) release, and caspase 3 (CASP3) activation which result in apoptosis. Knock down of HSPB1 attenuated the effects of NC009-1 on TBP/Q79 SH-SY5Y cells, suggesting NC009-1 exerts its neuroprotection through enhancing HSPB1. Our results demonstrate how indole compound NC009-1 reduces polyQ-induced aggregation and promotes neurite outgrowth to support its therapeutic potential in SCA17 treatment.

**PP30**

**Mitigation of progressive cerebellar symptoms of spinocerebellar ataxia by enhancing type 1 metabotropic glutamate receptor signaling**

Hirai H. ^1, 2^, Watanave M. ^1^, Konno A. ^1^

^1^ Department of Neurophysiology & Neural Repair, Gunma University Graduate School of Medicine, ^2^ Gunma University Initiative for Advanced Research

Corresponding author’s e-mail: hirai@gunma-u.ac.jp

Spinocerebellar ataxias (SCAs) are progressive, hereditary neurodegenerative diseases caused by a variety of gene defects, leading to movement disorder such as cerebellar ataxia. To date, no fundamental treatments for SCAs have been elucidated. Recent studies including ours have revealed that SCA type 1 (SCA1) and type 3 (SCA3) impair type 1 metabotropic glutamate receptor (mGluR1) and/or the closely associated signaling molecules in cerebellar Purkinje cell. Since the signaling pathway triggered by mGluR1 activation in Purkinje cell plays a pivotal role in coordinated movements and motor learning, repair of aberrant mGluR signaling in Purkinje cell may relieve cerebellar symptoms of SCAs. Indeed, we have recently shown that enhancement of mGluR1 signaling by baclofen, a clinically available GABAB receptor agonist, led to an improvement of motor performance in SCA1 mice and the improvement lasted ∼1 week after a single application of baclofen (J Physiol. 595(1):141-164, 2017). Here we present our recent results for SCA3, which show significant therapeutic influence of enhancing mGluR1 signaling by multiple approaches including pharmacological stimulation of retinoid-related orphan receptor-alpha, GABAB receptor and mGluR1.

**PP31**

**Cerebellar ataxia induced by oxytropis glabra poisoning in western Mongolian goats**

Shimada A. ^1^, Adilbish A. ^2^

^1^ Azabu University, Department of Environmental Pathology, Kanagawa, Japan ^2^ Institute of Veterinary Medicine, Ulaanbaatar, Mongolia

Corresponding author’s e-mail: a-shimada@azabu-u.ac.jp

Plants belonging to the Oxytropis and Astragalus genera are called locoweeds because they contain swainsonine, an indolizidine alkaloid of endophyte origin, and poisoning results in characteristic clinical and pathologic changes of locoism. Swainsonine inhibits lysosomal-mannosidase and Golgi mannosidase II, resulting in cellular vacuolation and degeneration in a variety of organs in multiple body systems including the reproductive, nervous, endocrine and immune systems; cerebral and cerebellar changes are responsible for the neurological signs of the poisoning.

In the last 5 years in western Mongolia, a neurological disorder and resultant economic loss have developed in goats, sheep, cattle and horses: association of the diseae with ingestion of Oxytropis glabra, a toxic plant, was suggested. Affected goats showed neurological signs, including ataxia, incoordination, hind limb paresis, fine head tremor, and nystagmus. Three goats, one with moderate clinical signs and the other two with severe clinical signs, were necropsied and examined to describe and characterize the histologic, immunohistochemical and ultrastructural lesions. Although no gross pathological changes were observed in these goats, microscopic examination of the cerebellum demonstrated degenerative changes in all these goats, such as vacuolar changes and loss of Purkinje cells, torpedo formation in the granular layer, increased number of spheroids in the cerebellar medulla, and loss of axons and myelin sheaths of Purkinje cells. The chemical analysis of the dried leaves, stems, and roots of the plant detected 0.02 – 0.05% (dry weight basis) 237, 454, and 484 μg/g DW of swainsonine, respectively. In the present goats, prominent lesions were observed in the cerebellum; this is different from those in the reported locoweed poisoning in domestic animals; cellular vacuolation was observed in a variety of organs in multiple body systems in addition to the cerebellum.

**PP32**

**Ataxia-related perturbation of human calcium channel biosynthesis**

FU Ssu-Ju. ^1^, JENG Chung-Jiuan. ^2^, PENG Yi-Jheng. ^1^,LEE Yi-Ching. ^1^, TANG Sung-Chun. ^3^ , HU Meng-Chun. ^1^, TANG Chih-Yung. ^4^

^1^Graduate Institute of Physiology, College of Medicine, National Taiwan University ^2^Institute of Anatomy and Cell Biology, National Yang-Ming University School of Medicine ^3^Department of Neurology, National Taiwan University Hospital ^4^Graduate Institute of Brain and Mind Science, College of Medicine, National Taiwan University

Corresponding author’s e-mail: d01441001@ntu.edu.tw

Objectives: Voltage-gated CaV2.1 channels play an essential role in regulating synaptic signaling. Mutations in the human CaV2.1 subunit are associated with the cerebellar disease episodic ataxia type 2 (EA2). Several EA2-causing mutants exhibit impaired protein stability and exert dominant-negative suppression of CaV2.1 wild-type (WT) protein expression via aberrant proteasomal degradation. Here, we delineate the protein degradation mechanism of human CaV2.1 subunit by identifying an E3 ubiquitin ligase RNF138.

Material and Methods: cDNAs of WT and mutant Cav2.1 transfected in to HEK293T cells for whole-cell patch and biochemistry analyses. Recombinant lentivirus was generated by co-transfecting cells with the packaging plasmid pCMV-Δ R8.91, the envelope plasmid pMD.G, and shRNA expressing constructs. Cortical neuron were isolated from SD rats.

Results: In neurons, RNF138 and CaV2.1 coexist in the same protein complex and display notable subcellular colocalization at presynaptic and postsynaptic regions. Overexpression of RNF138 promotes polyubiquitination and accelerates protein turnover of CaV2.1. Disrupting endogenous RNF138 function with a mutant (RNF138-H36E) or shRNA infection significantly upregulates the CaV2.1 protein level and enhances CaV2.1 protein stability. Disrupting endogenous RNF138 function also effectively rescues the defective protein expression of EA2 mutants. We propose that RNF138 plays a critical role in the homeostatic regulation of CaV2.1 protein level and functional expression and that RNF138 serves as the primary E3 ubiquitin ligase promoting EA2-associated aberrant degradation of human CaV2.1 subunits.

Conclusions: Protecting the human CaV2.1 subunit from excessive proteasomal degradation with specific interruption of endogenous RNF138 function may partially contribute to the future development of a novel therapeutic strategy for EA2 patients.

**PP33**

**Mitogen-activated protein kinase pathways are involved in Purkinje cell loss of spinocerebellar ataxia type 17 mice**

Lin C.W. ^1^, Chang Y.C. ^2^, Hsieh H.M. ^1^

^1^ Department of Life Science, National Taiwan Normal University ^2^ Department of Pharmacy, Taiwan Adventist Hospita

Corresponding author’s e-mail: hmhsieh@ntnu.edu.tw

The autosomal dominant inherited spinocerebellar ataxia type 17 (SCA17) is a neurodegenerative disease caused by the CAG/CAA expansion in the TATA-binding protein (TBP) gene. The neurological hallmark of the SCA17 disease is Purkinje cell loss and gliosis. Our laboratory has generated SCA17 transgenic mice that express human TBP with 109 CAG repeats (hTBP-109Q). These SCA17 mice showed ataxia and Purkinje cell loss, which recapitulate the patients’ phenotypes and are suitable for the study of SCA17 pathomechanism. In this study, we investigate the significance of MAPK pathways in SCA17 using the cerebellar primary culture and SCA17 mice. We found that the degeneration of Purkinje cells occurs in the SCA17 transgenic mice since 6 weeks old. The presence of gliosis and TBP nuclear aggregation was also observed since then. Moreover, we found that a higher expression of pERK may contribute to gliosis in the SCA17 mouse cerebellum. We also found that the expression level of cleaved caspase3 was significantly increased in the 6 week-old SCA17 mouse cerebellum. The expression level of pp38 was increased in the 8 week-old SCA17 mouse cerebellum. To ascertain the relationship between the MAPK pathways and neuronal apoptosis, we examined the upstream and downstream targets of these two pathways. We confirm the association between MAPK pathway and Purkinje cell apoptosis by SCA17 primary culture. Our study might provide a new therapeutic strategy for the SCA17 disease.

**PP34**

**Pathogenesis and future therapy of spinocerebellar ataxia type 31 (SCA31)**

Kinya Ishikawa ^1,2^, Taro Ishiguro ^1^, Nozomu Sato ^1^, Morio Ueyama ^3,4^, Yuji Hashimoto ^1^, Takeru Honda ^5^, Soichi Nagao ^5^, Takanori Yokota ^1^, Nicolas Charlet-Berguerand ^6^, Christopher E. Pearson ^7^, Yoshitaka Nagai ^3,4^, Hidehiro Mizusawa ^1^

^1^ Department of Neurology and Neurological Science^2^ Department of Personalized Genomic Medicine for Health, Graduate School, Tokyo Medical and Dental University, 1-5-45 Yushima, Bunkyo-ku, Tokyo 113-8519, Japan

^3^ Department of Degenerative Neurological Diseases, National Institute of Neuroscience, National Center of Neurology and Psychiatry, 4-1-1 Ogawa-Higashi, Kodaira, Tokyo 187-8502, Japan

^4^ Department of Neurotherapeutics, Osaka University Graduate School of Medicine, 2-2 Yamadaoka, Suita, Osaka 565-0871, Japan

^5^ Laboratory for Motor Learning Control, RIKEN Brain Science Institute, 2-1 Hirosawa, Wako, Saitama 351-0198, Japan

^6^ Institut de Génétique et de Biologie Moléculaire et Cellulaire, University of Strasbourg, Illkirch 67400, France

^7^ Department of Genetics, The Hospital for Sick Children, Peter Gilgan Centre for Research & Learning, 686 Bay Street, Toronto, Ontario M5G 0A4, Canada

Corresponding author’s e-mail: piconuro@tmd.ac.jp

Background: Spinocerebellar ataxia type 31 (SCA31) is one of the most common SCAs in Japan showing slowly progressive pure cerebellar ataxia. Clinical assessment is critical in future development of fundamental therapy, and we are using prism adaptation system for rating cerebellar dysfunction. This disease is caused by the presence of a complex penta-nucleotide repeat that consists of (TGGAA)n in an intronic region shared by two genes, BEAN1 (brain expressed, associated with NEDD4) and TK2 (thymidine kinase 2). The critical portion (TGGAA)n is expressed as (UGGAA)n in BEAN1 direction forming abnormal RNA structures (RNA foci) in SCA31 patients’ Purkinje cells.

Objectives: To elucidate how (UGGAA)n underlies SCA31 pathogenesis.

Results: When the SCA31 repeat containing (UGGAA)n was expressed in Drosophila compound eyes, various morphological disruptions were observed accompanied by RNA foci, the degree matching with the length of (UGGAA)n, the level of (UGGAA)n expression, and with the amount of RNA foci. A penta-peptide repeat protein predicted to be translated from (UGGAA)n was also expressed in SCA31 fly eyes, again in an agreement with the expression level. In contrast, a complex penta-nucleotide repeat lacking (UGGAA)n did not show obvious degeneration of eyes, nor RNA foci.

RNA pull-down assay followed by proteomic study disclosed a number of potential proteins that bind with (UGGAA)n. Among these, TDP-43 was confirmed to bind directly with (UGGAA)n by western blotting. Immunohistochemistry also showed that TDP-43 co-localized with (UGGAA)n-containing RNA foci in patients’ Purkinje cells as well as with RNA foci in SCA31 flies. Interestingly, the toxicity of (UGGAA)n was dampened when the SCA31 flies were crossed with the ones that overexpress TDP-43. TDP-43 directly suppressed (UGGAA)n-aggregation under atomic force microscopy, and circular dichroism (CD)- spectroscopy disclosed that TDP-43 altered (UGGAA)n structure upon binding. TDP-43 reduced not only RNA foci but also the amount of penta-peptide repeat protein.

Conclusion: These suggest that TDP-43 play a critical role in (UGGAA)n-mediated toxicity through an RNA chaperone activity.

**PP35**

**Mutations in VPS13D lead to a new recessive ataxia with spasticity and mitochondrial defects**

Eunju Seong^1^, PhD, Ryan Insolera^2^, PhD, Marija Dulovic^3^, PhD, Erik-Jan Kamsteeg^4^, PhD, Norbert Brüggemann^5^, MD, Tamison Jewett^8^, MD, Vikram Shakkottai ^10^, MD PhD, Christine Klein^3^, MD, Catherine Collins^2^, PhD, Katja Lohmann^3*,^ PhD, Bart P. van de Warrenburg ^11*,^ MD PhD, Margit Burmeister^1,6,7,12^*, PhD

^1^Molecular & Behavioral Neuroscience Institute, University of Michigan, Ann Arbor, MI 48109, USA

^2^Department of Molecular, Cellular, and Developmental Biology, University of Michigan, Ann Arbor, MI 48109, USA ^3^Institute of Neurogenetics, University of Lübeck, Germany ^4^Department of Human Genetics, Radboud University Medical Centre, Nijmegen, The Netherlands ^5^Department of Neurology, University of Lübeck, Germany ^6^Department of Human Genetics, University of Michigan, Ann Arbor, MI 48109, USA ^7^Department of Computational Medicine & Bioinformatics, University of Michigan, Ann Arbor, MI 48109, USA^8^Department of Pediatrics, Section on Medical Genetics, Wake Forest School of Medicine, Winston-Salem, North Carolina, USA^9^Department of Clinical Genetics, Erasmus MC, Rotterdam, the Netherlands

^10^Departments of Neurology and of Molecular and Integrative Physiology, University of Michigan, Ann Arbor, MI 48109, USA

^11^Department of Neurology, Donders Institute for Brain, Cognition and Behaviour, Radboud University Medical Center, Nijmegen, The Netherlands^12^Department of Psychiatry, University of Michigan, Ann Arbor, MI 48109, USA

Objectives: To identify novel causes of recessive ataxias, including spinocerebellar ataxia with saccadic intrusions, spastic ataxias and spastic paraplegia.

Methods: In an international collaboration, we independently performed exome sequencing in seven families with recessive ataxia and/or spastic paraplegia. To evaluate the role of VPS13D mutations, we evaluated a Drosophila knock-out model and investigated mitochondrial function in patient-derived fibroblast cultures.

Results and Conclusions: Exome sequencing identified compound heterozygous mutations in VPS13D on chromosome 1p36 in all seven families. This included a large family with 5 affected siblings with spinocerebellar ataxia with saccadic intrusions (SCASI), or spinocerebellar ataxia, recessive, type 4, SCAR4. Linkage to chromosome 1p36 was found in this family with a LOD score of 3.1. The phenotypic spectrum in our 12 patients was broad. Although most presented with ataxia, additional or predominant spasticity was present in 5 patients. Disease onset ranged from infancy to 39 years, and symptoms were slowly progressive and included loss of independent ambulation in 5. All but two patients carried a loss-of-function (nonsense or splice site) mutation on one and a missense mutation on the other allele. Knock-down or removal of Vps13D in Drosophila neurons led to changes in mitochondrial morphology and impairment in mitochondrial distribution along axons. Patient fibroblasts showed altered morphology and functionality including reduced energy production. Our study demonstrates that compound heterozygous mutations in VPS13D cause movement disorders along the ataxia-spasticity spectrum, making VPS13D the fourth VPS13 paralog involved in neurological disorders.

(in press in Annals of Neurology, 2018)

**PP36**

**Alcohol-related ataxia: clinical, radiological and neurophysiological characteristics**

Shanmugarajah P.D. ^1^, Gleeson D.C. ^2^, Grünewald R.A. ^1^, Hadjivassiliou M. ^1^, Hoggard N. ^3^

^1^ Academic Department of Neurosciences, Royal Hallamshire Hospital and University of Sheffield, Sheffield, UK ^2^ Academic Department of Hepatology, Royal Hallamshire Hospital and University of Sheffield, Sheffield, UK ^3^ Academic Unit of Radiology, University of Sheffield, Sheffield, UK

Corresponding author’s e-mail: p.d.shanmugarajah@sheffield.ac.uk

Introduction: Alcohol-related ataxia is one of the commonest acquired causes of cerebellar ataxia. The mechanisms remain to be explored.

Objective: We aim to evaluate the clinical, radiological and neurophysiological characteristics of alcohol-related ataxia.

Material and methods: Patients with a history of chronic alcohol dependence diagnosed with alcohol-related ataxia between 2014 and 2017 at the Sheffield Ataxia Centre, UK. All patients were investigated for other causes of ataxia and excluded if an alternative cause was found. Patients underwent a 3T MRI brain and cerebellar spectroscopy, and when clinically indicated, a neurophysiological assessment.

Results: We reviewed 34 patients with alcohol-related ataxia. Median age at presentation was 58 years (range 29 to 83 years). All patients had poor balance symptoms and 15 (44%) patients had limb sensory symptoms. Eighty eight percent (30/34) had mild severity ataxia (walking independently/with 1 walking aid). Sixty five percent (22/34) had nystagmus/broken pursuit eye movements and 16/34 (47%) had a cerebellar tremor. All patients had gait ataxia and 19/34 (56%) had limb ataxia. Depressed/absent ankle jerks were present in 23/34 (68%) patients. Brain atrophy (generalised/fronto-parietal) on MRI was present in 25/34 (74%) patients. Cerebellar atrophy was present in 29/34 (85%) and abnormal cerebellar MR spectroscopy (vermis NAA/Cr ≤ 0.95 and/or hemisphere NAA/Cr ≤ 1.00) was seen in 26/34 (76%) of patients with predominantly vermian dysfunction. Vermian NAA/Cr ranged from 0.45 to 1.27 (median 0.87) and hemispheric NAA/Cr ranged from 0.61 to 1.32 (median 0.92). Twelve patients had nerve conduction studies of which 4 had a length dependent sensory-motor axonal peripheral neuropathy.

Conclusions: Alcohol-related ataxia is associated with cerebellar dysfunction with a predilection for the vermis. The high prevalence of brain atrophy suggests more widespread brain pathology in addition to cerebellar ataxia.

**PP37**

**Prominence of cerebellar pathways in brain white-matter abnormalities in Friedreich ataxia: The IMAGE-FRDA study**

Selvadurai L.P. ^1^, Corben L.A. ^1,2,3^, Stagnitti M.R. ^1^, Storey E. ^4^, Egan G.F. ^1,5^, Delatycki M.B. ^2,6^, Georgiou-Karistianis N. ^1^, Harding, I.H. ^1^

^1^ Monash Institute of Cognitive and Clinical Neurosciences/School of Psychological Sciences, Monash University, Clayton, Australia^2^ Bruce Lefroy Centre for Genetic Health Research, Murdoch Childrens Research Institute, Parkville, Australia^3^ Department of Paediatrics, The University of Melbourne, Parkville, Australia^4^ Department of Medicine, Monash University, Prahran, Australia^5^ Monash Biomedical Imaging, Monash University, Clayton, Australia^6^ Clinical Genetics, Austin Health, Heidelberg, Australia

Corresponding author’s e-mail: louisa.selvadurai@monash.edu

Objectives: Friedreich ataxia (FRDA) is a recessively inherited degenerative disorder. White-matter abnormalities are observed in cerebellar pathways and the cerebrum, although the relative size of effects across the brain and their pathophysiology remain unclear. Better characterisation of white-matter abnormalities is important for developing theoretical models and biomarkers. This study aimed to characterise white-matter abnormalities in FRDA, their clinical significance, and relationships between different white-matter imaging measures.

Methods: 31 individuals with FRDA and 36 healthy controls undertook brain Magnetic Resonance Imaging. Diffusion-tensor, magnetisation-transfer, and T1-weighted imaging produced whole-brain maps of overall white-matter integrity, myelin integrity, axonal integrity, and white-matter volume. Between-group comparisons were conducted. Each white-matter metric was correlated with clinically-measured disease severity and disease onset age. Correlations between metrics were investigated.

Results: Individuals with FRDA showed widespread white-matter deficits, particularly in diffusion metrics. The largest deficits occurred in the cerebellar peduncles. Greater deficits correlated with more severe clinical presentation and earlier onset, primarily in cerebellar and corticospinal pathways. Group differences in overall white-matter integrity were closely associated with those in diffusion-based myelin integrity but relatively independent of other measures.

Conclusions: FRDA involves widespread white-matter deficits in the brain, most apparent in cerebellar pathways. Deficits are more severe in individuals with earlier disease onset and greater disease severity, which may indicate the contribution of both developmental and degenerative changes. Myelin-related deficits may largely drive these abnormalities. Different white-matter metrics provide largely independent findings, and therefore all should be examined as potential biomarkers.

**PP38**

**Perturbation in Magnetic Resonance Images (MRIs) from Cerebellum Target Stereotactic Radiosurgery (SRS)**

Kaile Li

John R Marsh Cancer Center, Hagerstown, USA

Corresponding author’s e-mail: goolkl@gmail.com

Introduction: Stereotactic Radiosurgery (SRS) is an efficacy procedure in treatment of brain disease. The complicated SRS procedure includes simulation, target definition, treatment planning, target localization and dose delivery. The external accuracy verification of whole procedure have been investigated with different quality assurance methodologies. However, the final estimation of SRS procedure should be reflected in the disease lesions inside the patient, and this could be done by employing different imaging modalities at different temporal points but challenges exist in abstracting the weak signal due to radiation in the images. Therefore, in this study, a method was used to estimate the perturbation information in MRIs at different temporal points after a cerebellum target SRS.

Methods and Materials: A cerebellum target was under a SRS with a single ARC small aperture cone on a Linac machine from Varian Medical system. A series of MRIs in different temporal points have been attained, the temporal range was 0 month, 3 months, 6 months and 9 months. The volume of interested scans were defined by the isodose volume in the dosimetric plan, which included target volume, and isodose volumes which were at different isodose levels including 100%, 90%, 75%, 60%, 30% and 15% of prescription dose. Through image fusion method, these volumes of interest were defined in the MRIs through the function of copy structures to registered image. Then structure property function to attain the structure statistics including minimum Hounsfield Unit (HU), maximum HU, mean HU and standard deviation (SD) of HU inside the volume of interested. Vectors were used to represent the separate volumes of interest and corresponding statistics in HU. A relative percentage difference method, which was defined to be the ratio between the differences of SD and mean SD divided by the mean SD to separate the technical variation from imaging procedure.

Result: For the selected volumes of interest, the mean SD HUs were 20.8, 20.4, 24.1, and 26.21 for T1 MRIs, and was 35.2, 17.4, 31.9, and 37.1 for T1 MRIs with contrast. And the least difference in SD HU vector elements was at 3 month, and the average absolute SD HUs was about 3 in magnitude. Moreover, the relative percentage difference showed time-spatial vector pattern with special characteristics.

Conclusions: Some significant HU variation can be seen from T1 and T1 with contrast MRIs in temporal and volume discrete matrix. Data analysis could be further improved by eliminating the uncertainty due to technical inconsistency, and similar investigation approach could be applied to the MRIs acquired right after radiation irradiated for SRS.

**PP39**

**Ubiquitin proteasome pathway modulation as therapeutic strategy for polyQ-mediated spinocerebellar ataxia type 3**

Chen I.C. ^1^, Chang C.N. ^2^, Chen W.L. ^1^, Lin T.H. ^2^, Chao C.Y. ^1^, Wu Y.R. ^1^, Lee-Chen G.J. ^2^, Chen C.M. ^1^

^1^ Department of Neurology, Chang Gung Memorial Hospital, Chang Gung University College of Medicine, Taipei 10507, Taiwan^2^ Department of Life Science, National Taiwan Normal University, Taipei 11677, Taiwan

Corresponding author’s e-mail: ichen910@gmail.com

Autosomal dominant spinocerebellar ataxias (SCAs) are caused by the abnormal expansions of CAG trinucleotide repeats and associated polyglutamine (polyQ) tract. Accumulation of aggregated disease proteins is a common feature of polyQ diseases, leading to progressive neuronal dysfunction and degeneration. SCA type 3 (SCA3), the most common form of SCA worldwide, is characterized by a CAG triplet expansion in chromosome 14q32.1 ATXN3 gene. As accumulation of the mutated polyQ protein is a possible initial event in the pathogenic cascade, clearance of aggregated protein by ubiquitin proteasome system (UPS) is supposed to inhibit downstream detrimental events to suppress neuronal cell death. In this study Chinese herbal medicine (CHM) extracts were examined for enhancing proteasome activity, inhibiting polyQ aggregation and neuroprotection using GFPu and ATXN3/Q75-GFP 293/SH-SY5Y cells. Among the 14 tested extracts, 8 displayed increased proteasome activity which was confirmed by 20S proteasome activity assay and analysis of ubiquitinated and fused GFP proteins in GFPu cells. All the 8 extracts displayed good aggregation-inhibitory potential when tested in ATXN3/Q75-GFP 293 cells. Among them, neuroprotection effects of 5 selected extracts were further confirmed by analyses of polyQ aggregation, neurite outgrowth, caspase 3/proteasome activities, and ATXN3-GFP/ubiquitin/Bcl-2/Bax protein levels in neuronal differentiated ATXN3/Q75-GFP SH-SY5Y cells. Finally enhancement of proteasome, anti-oxidation and neuroprotection of active constituents catalpol, puerarin and daidzein and Rehmannia glutinosa and Pueraria lobata extracts were affirmed in GFPu or ATXN3/Q75 SH-SY5Y cells. This study may have therapeutic implication in polyQ-mediated disorders.

**PP40**

**Developing a clinically meaningful instrumented measure of upper limb function in Friedreich ataxia.**

Corben L.A ^1,2,3^, Tai G ^1^, Szmulewicz D ^4,5,6,^ Horne M.K ^6^, Pathirana P.N ^7^, Delatycki M.B ^1,2,3,8^

^1^Bruce Lefroy Centre for Genetic Health Research, Murdoch Childrens Research Institute, Parkville, Vic., Australia^2^School of Psychological Sciences, Monash University, Clayton, Vic., Australia^3^Department of Paediatrics, University of Melbourne, Parkville, Vic., Australia^4^Royal Victorian Eye and Ear Hospital, East Melbourne, Vic., Australia ^5^Alfred Hospital, Prahran, Vic., Australia^6^Florey Institute, Parkville, Vic., Australia^7^School of Engineering, Deakin University, Geelong, Vic., Australia^8^Victorian Clinical Genetics Service, Parkville, Vic., Australia

Corresponding author’s e-mail: louise.corben@vcgs.org.au

Objective. Friedreich ataxia (FRDA) has a significant effect on upper limb function. The most common measure of upper limb function in FRDA is the Nine Hole Peg Test (9HPT) however the capacity for the 9HPT to reflect functional capacity is uncertain. This study aimed to 1) identify the functional upper limb tasks that individuals with FRDA find most challenging and using these results develop a novel measure of upper limb function and, 2) identify if this measure differentiates the upper limb function in individuals with ataxia from control subjects.

Method. We analysed the upper limb component of the Friedreich Ataxia Impact Scale (FAIS), performance on the Jebsen Taylor Hand Function Test (JHFT) and 9HPT in 120 individuals with FRDA. Based on these data we developed a new upper limb measure, the BioKin wireless motion capture device, which yields a composite score based on three dimensional kinematic parameters captured during a vital upper limb task for individuals with FRDA: the pre-oral phase of eating.

Results. The composite score captured via the BioKin-WMS in 26 adults and children with FRDA was significantly different from that obtained from the 12 matched control subjects (t(36)= 6.26, p < 0.001). There were significant correlations between the composite score and clinical parameters: age at disease onset (r = 0.86, p < 0.001), disease duration (r = 0.91, p < 0.001), GAA1 (r = 0.91, p < 0.001) and GAA2 (r = 0.96, p < 0.001), confirming the capacity of the composite score to reflect disease status.

Conclusion: We have systematically identified a functional task that has provided the genesis for development of a measure of upper limb dysfunction in people with FRDA, relevant to both children and adults and can be completed by individuals who are unable to complete the 9HPT. As such, it has the potential to be applicable to most individuals with ataxia across the disease trajectory which will ensure that individuals with severe ataxia can be included in clinical trials.

**PP41**

**Cerebellum, stability-balance test, and combinatory Spectrum dosages(NP=P)**

hsihchia.hsieh, Pei-gin-Heieh^1^

^1^ National Chung-cheng University

Corresponding author’s e-mail: hsihchia.hsieh@gmail.com

1.The target is to find the causality development of abnormality at time T(t)=t>(or<)n cell evolution , and therapies which is new-frontier types of combinatorial treatment dosages, aiding by tomography, physiology and numerical analysis with overlapping boundaries. 2.Method of anatomy is: Non-deterministic polynomials(NP=P), computing the dynamic equilibrium which is a shifting stability and balance in exercise with a faster speed of connections for cell growth than existing treatments.

3.1:Findings:The stringent authority uses the laser and x-ray tomography, localizing the sensitivity and specificity of abnormal mutation and pains, allowing thepatients to exercises within finite steps and finite volume of energy;

3.2: the NP=P computes precision differences through overlapping the images of the normal and abnormal brain images,

3.3:NP=P is a combinatory treatment dosage, using animal models for diagnosis of abnormality; specific recovery times, and patent treatment mechanism, theory and experiment are consistent with electronic and magnetic interactions at the cell junctions’ 3.4:Disorder is reflected as too long or two short transcription errors; the cut-off the cell junction is diagnosed through the rotating cycle of inverse-Fourier transform.

3.5 Results and open problems: the transitions from disorder to the normal order are non-trivial dynamic equilibrium in terms of neurology and cost;

3.6:exercise is rotation cycles in metaborithm of hydrophobic-hydro-physics, absorption and diffusion; the regulation of cerebellum is the constraint of brain’s local and global body, like photosynthesis, in inter-discipline sciences for patients’ phase III trials.

3.7 major contribution is the geometric index of diagnosis of images of normal and abnormal images, which is consistent pH base/acid boundary and blood tests of viscosity, which solves the matrix of eigenvalues and vectors in finite volumes for integration and differentiation in discrete and continuous physiology and anatomy.

**PP42**

**Objective assessment and rating of cerebellar ataxia measured by a depth sensor**

Honda T. ^1,2,3^, Mitoma H. ^4^, Taguchi T. ^4^, Terashi H. ^4^, Aizawa H. ^4^, Mizusawa H. ^3^, Manto M. ^5^, Kakei S. ^1^

^1^ Tokyo Metropolitan Institute of Medical Science, Motor Disorders Project, Tokyo, Japan^2^ RIKEN, Center of Brain Science, Laboratory for Behavioral Genetics, Saitama, Japan^3^ National Center of Neurology and Psychiatry, Tokyo, Japan^4^ Tokyo Medical University, Tokyo, Japan

^5^ UMons, Service des Neurosciences, Mons, Belgium

Corresponding author’s e-mail: honda-tk@igakuken.or.jp

The severity of motor symptoms induced by cerebellar diseases is quantified using various clinical scales, such as the International cooperative ataxia rating scale (ICARS) and the Scale for the assessment and rating of ataxia (SARA). Since the assessment of grading can be subjective, differences of scaling in overall assessments are unavoidable, especially among non-expert examiners. Thus, several types of quantitative clinical tests on cerebellar ataxias have been dependent on examiner’s experiments. Here, we developed to measurements of the whole body movements and proposed a new method to evaluate the motor functions at the level of movement kinematics by using the Kinect v2 sensor (Microsoft Inc.). It is very easy for participants to use our system anywhere there are a Kinect v2 sensor and a windows computer connected with internet. The reference regions, which reproduce a body image, include the head, spine (shoulder, thoracic, lumbar level), hand (index finger), elbow, shoulder, heel, knee, and the pelvis. Second, using our newly developed algorithm, the position data from the reference regions are transformed into parameters that are used for assessment in SARA. We digitally revealed unbalance during walking, stance and sitting, overshoot and undershoot during finger chase, terminal tremor, and dysrhythmic during fast alteration finger movements in 5 cerebellar patients (two CCA, one SCA6, and two MSA-C). We simultaneously found abnormal scattering of all points of body. The results suggest that many aspects of cerebellar ataxias, such as clumsiness, irregularity and inaccuracy, can be objectively identified and quantified by our system. This system may connect patients with medical doctors on-line and may ubiquitously provide a practical test of motor control. We are going to connect the system with a cloud database system (e.g., Microsoft Azure) so that the system will truly put remote diagnosis into practice.

**PP43**

**Hyperbaric oxygen ameliorates the neurobehaviors of spinocerebellar ataxia type 17 mice**

Chiang M.K. ^1^, Lin C.W. ^1^, Lin K.H. ^2^, Hsieh H.M.^1^

^1^ Department of Life Science, National Taiwan Normal University ^2^ Thoughtful animal Hospital

Corresponding author’s e-mail: hmhsieh@ntnu.edu.tw

Spinocerebellar ataxia type 17 (SCA17) is an autosomal dominant inherited neurodegenerative disease. Patients with progressive cerebellar atrophy, ataxia, epilepsy, and dementia. SCA17 is caused by abnormal amplification of CAG/CAA trinucleotide on the TATA-box binding protein (TBP) gene, when this trinucleotide is repeated for more than 42 times. However, no drug has the significant effect on the treatment of SCA17 until now. Hyperbaric oxygen (HBO) therapy is a painless, non-invasive treatment that is used for carbon monoxide poisoning, arterial embolism, traumatic ischemia, burns, and decompression sickness. Patients are treated in a cabinet with high atmospheric pressure and 100% oxygen for a period of time. It is reported HBO reduces the inflammatory response and hypoxia in tissue, and improves microvascular proliferation. In this study, 6-month-old SCA17 transgenic mice were HBO treated and behavior tests were performed before and after treatment. Behavior tests include the open-field, Y-maze, footprint, and rota-rod. After HBO treated for 14 times, the results showed that HBO therapy had no effect in anxiety and gait ataxia in the open-field and footprint tests. While the Y-maze and rotarod tests showed HBO significantly improved the short-term memory and motor-coordination. After the monthly behavioral experiment, we learned that the duration of this HBO effect can be lasted for 3 months. Therefore, we believed that HBO has therapeutic potential for SCA17 mice. In the future, we will conduct HBO therapy in older animals, and pathological analysis will be characterized to elucidate the molecular mechanism to assess HBO therapy effect.

**PP44**

**Combination of AAV-PHP.B with a cell type-specific promoter can express a transgene in a cell type-specific manner throughout the brain.**

Ayumu Konno, Yoichiro Shinohara, Keisuke Nitta, Chiaki Hoshino, Hirokazu Hirai

Dept Neurophysiol & Neural Repair, Gunma Univ, Gunma, Japan

Corresponding author’s e-mail: konnoa@gunma-u.ac.jp

Adeno-associated virus (AAV) vectors are powerful tools for gene transfer vehicles in living animals. Since AAV vector can infect and express a transgene in both dividing and non-dividing cells in the central nervous system (CNS), it is widely used in neuroscience research. Recently, Deverman and his colleagues reported AAV-PHP.B, a capsid variant of AAV9, which exhibited a great permeability of the blood brain barrier (BBB) in mice. The intravenous injection of AAV-PHP.B in adult mice can efficiently transduce both glial cells and neurons. Since CNS is comprised of diverse types of cells, cell type-specific transgene expression is a useful approach for investigating complex brain functions. We recently reported cell type-specific promoters suitable for viral vectors, especially for AAV vector (Shinohara et al, PLoS ONE, 2016; Shinohara et al, Cerebellum, 2017; Nitta et al, Mol Ther MCD, 2017). We thought that combination of AAV-PHP.B with a cell type-specific promoter allowed us to express a transgene in a cell type-specific manner throughout the brain. To prove this, we intravenously injected the AAV-PHP.B expressing GFP under the control of the NSE (neuron-specific enolase) or Gfap (glial fibrillary acidic protein) promoter to mice. Three weeks after the viral injection, we observed neuron-specific or astrocyte-specific 　GFP expression throughout the CNS. Next, we mixed AAV-PHP.B expressing GFP under the control of Gfap promoter and those expressing RFP under the control of the NSE promoter, and the mixture was intravenously injected to mice. The results showed that astrocytes and neurons were separately labeled with GFP and RFP, respectively. In addition, we have different cell-type-specific promoters, and are going to present results obtained using AAV-PHP.B carrying those promoters.

**PP45**

**Mycophenolate as treatment of autoimmune ataxias**

Hadjivassiliou M. ^1^, Grunewald R.A. ^1^, Shanmugarajah P. ^1^, Sarrigiannis P. ^1^, Zis P. ^1^, Hoggard N. ^2^

^1^ Academic Department of Neurosciences, Sheffield Teaching Hospitals NHS Trust^2^ Academic department of Neuroradiology, Univesrity of Sheffield

Corresponding author’s e-mail: m.hadjivassiliou@sheffield.ac.uk

Objectives: To evaluate the effectiveness of immunosuppression in patients with autoimmune cerebellar ataxias.

Material and methods: Patients attending the Sheffield Ataxia Centre were suspected of having autoimmune ataxia if 1) all other causes of ataxia had been excluded 2) cerebellar involvement suggested primarily vermian dysfunction (clinically and on MR spectroscopy of the cerebellum) 3) patients were positive anti-GAD antibodies and/or had a history of autoimmune diseases. We excluded patients with paraneoplastic cerebellar degeneration and patients with gluten ataxia who responded to gluten-free diet. Patients underwent baseline MR spectroscopy (MRS) of the vermis. Mycophenolate was introduced at 500 mg twice daily and the dose was increased to 1 gm twice daily at 1 month. Patients were re-assessed on a 4 monthly basis. Repeat MRS was performed after treatment.

Results: We treated 17 patients. Eight patients with autoimmune ataxia, not on treatment were used as controls. In the treatment group, 9 patients had primary autoimmune cerebellar ataxia (PACA), 4 had ataxia with anti-GAD antibodies and 4 had gluten ataxia unresponsive to gluten free diet and persistent enteropathy. In the untreated group, 5 patients had ataxia with anti-GAD antibodies and 3 had PACA. Mean age of the treatment group was 63 (range 37-83) and of the untreated group was 66 (range 43-82). The mean time between baseline and second MRS scan was 18 months (range 6 to 30) in both groups. The mean NAA/Cr ratio in the treatment group increased from 0.78 to 0.93 and in the non-treatment group decreased from 0.86 to 0.68 (p < 0.0001, 2 tailed t test). All patients in the treatment group demonstrated increased NAA/Cr whilst all in the non-treated group demonstrated decrease NAA/Cr ratio. The MR spectroscopy changes were associated with clinical improvement in the treatment group and deterioration in the untreated group.

Conclusions: Mycophenolate appears effective as treatment of autoimmune ataxia.

**PP46**

**Minimal Purkinje Cell-Specific L7 Promoter Virally Available for Rodents and Non-human Primates**

Matsuzaki Y. ^1^, Nitta K. ^1^, Konno A. 1, Hirai H. ^1^

^1^ Gunma university, department of Neurophysiology and Neural Repair, Maebashi, Japan

Corresponding author’s e-mail: y.matsuzaki@gunma-u.ac.jp

Cerebellar Purkinje cells play a pivotal role in cerebellar functions. The cerebellar Purkinje cell-specific L7 promoter is widely used to express transgenes in Purkinje cells. The L7 promoter comprises large size (3 kb) of mouse Pcp2 gene and exceedingly weak promoter activity. Although it works well in generation of transgenic mice, the long size and weak promoter strength are unfavorable for viral vectors. Here, we attempted to identify minimal region of the L7 promoter that maintains Purkinje cell specificity and promoter strength. In vitro and in vivo experiments using lentiviral vectors showed that the 0.8-kb region (named L7-6) upstream of the transcription initiation codon in the first exon of Pcp2 gene was alone sufficient as a Purkinje cell-specific promoter, presenting a far stronger promoter activity over the original 3-kb L7 promoter with a significant specificity to Purkinje cells. Intravenous injection of an adeno-associated virus vector (AAV-PHP.B) that are highly permeable to the blood-brain barrier confirmed the Purkinje cell specificity of the L7-6 promoter in the CNS. The features of the L7-6 promoter were also preserved in the marmoset, a non-human primate. The high sequence homology of the L7-6 promoter among mouse, marmoset, and human suggests the preservation of the promoter strength and Purkinje cell specificity also in humans. These findings suggest that L7-6 promoter will facilitate the cerebellar research targeting the pathophysiology and gene therapy of cerebellar disorders.

**PP47**

**Chemogenetic interrogation of the role of cerebellum lobules IV-V in social cognition**

Chao O.Y. ^1^, Monley B. ^2^, Yunger R. ^3^, Yang Y.M. ^1^

^1^ Department of Biomedical Sciences, University of Minnesota Medical School, Duluth MN, USA^2^ Medical School, University of Minnesota Duluth, Duluth MN, USA^3^ Swenson College of Science-Engineering, University of Minnesota Duluth, Duluth MN, USA

Corresponding author’s e-mail: ymyang@d.umn.edu

The role of cerebellum has been evidenced not only in sensorimotor functions but in learning, memory and social cognition. Although the posterior cerebellum lobules VI-VII involve in social functions, how the anterior lobules IV-V affect social cognition remains enigmatic. Patients and animals with damaged lobules IV-V show sensorimotor deficits, which confounds their social performance. To circumvent this limitation, we hypothesized that excitation of the lobules IV-V would bypass the sensorimotor deficits induced by lesion or inhibition and provide its functional information. To this end, we applied chemogenetics by transferring excitatory DREADD, AAV-hSyn-hM3Dq-mCherry, or a control AAV-hSyn-GFP, into the lobules IV-V of C57BL/6 mice. Three weeks later, animals were tested by elevated plus maze, open field, novel object memory, social recognition memory, rotarod and freely social interaction test. Clozapine-N-oxide (0.8 mg/kg, i.p.) was administrated 40 min prior to each test in both groups. In the open field, hM3Dq group showed more distance travelled than GFP group, while comparable performance was found by a rotarod. Both groups showed intact object recognition memory. In social recognition, both groups showed more time sniffing a stranger mouse contained by a cup than an empty cup in the learning trial. In the test trial, GFP group sniffed more toward a novel stranger than the familiar one, but hM3Dq group did not. For freely social interaction, hM3Dq group exhibited less interaction than GFP group. In vitro electrophysiology and immunostaining were employed to confirm the neuronal effects of hM3Dq receptors and the location of transfected neurons in the lobules IV-V. Our results demonstrate that chemogenetic excitation of the lobules IV-V impairs social recognition and freely social interaction, without affecting motor coordination and learning. The cerebellum lobules IV-V may involve in the brain networks associated with social cognition.

**PP48**

**Granule cell precursors in the lateral cerebellum are preferentially sensitive to elevated sonic hedgehog signaling and formation of medulloblastoma**

Tan I.L. ^1,2^, Wojcinski A. ^1^, Rallapalli H. ^3^, Lao Z. ^1^, Sanighrajka R.M. ^1,2^, Stephen D. ^1^, Volkova E. ^3^, Korshunov A. ^4^, Remke M. ^5^, Taylor M.D. ^5,6^, Turnbull D.H. ^3^ and Joyner A.L. ^1,2^

^1^ Developmental Biology Program, Sloan Kettering Institute, New York, NY, 10065^2^ Biochemistry, Cell and Molecular Biology Program, Weill Graduate School of Medical Sciences of Cornell University, New York, NY, 10065^3^ Skirball Institute of Biomolecular Medicine and Department of Radiology, NYU School of Medicine, New York, NY, 10016^4^ Clinical Cooperation Unit Neuropathology, German Cancer Research Center (DKFZ), and Department of Neuropathology, University Hospital, Heidelberg, Germany^5^ The Arthur and Sonia Labatt Brain Tumour Research Centre, Hospital for Sick Children, Toronto, Canada^6^Division of Neurosurgery, Department of Surgery, University of Toronto

Corresponding author’s e-mail: ilt2002@med.cornell.edu

Objective: Granule cell precursors (GCPs) are a sonic hedgehog (SHH)-dependent progenitor population in the developing cerebellum and the main cell of origin for the SHH subgroup of medulloblastoma (MB). Unlike other subgroups of MB, SHH-MBs occur preferentially in the lateral cerebellum (hemispheres) and have four main driver mutations. We studied whether the timing or type of mutation affects tumor location and identified factors influencing SHH-MB progression.

Methods: We analyzed the association between type of mutation and tumor location in 38 SHH-MB patient samples. To generate sporadic mouse models of SHH-MB, inducible recombinases were used to express a constitutive activate SMO receptor (SmoM2) or delete Ptch1 in only scattered GCPs. Tumor location, expression profiles and GCP behaviors were analyzed in the models.

Results: Our analysis of patient data indicates that adult tumors with SMO mutations form more specifically in the hemispheres than those with PTCH1 mutations. Using sporadic mouse models, we found that regardless of the number of GCPs mutated, timing or type of mutation, tumors developed almost exclusively in the hemispheres with SmoM2-mutants showing a stronger specificity. We further uncovered that GCPs in the hemispheres are more susceptible to high level SHH signaling compared to GCPs in the medial cerebellum (vermis), as more mutant cells in the hemisphere remain undifferentiated and show increased tumorigenicity when transplanted. We also identified location-specific gene expression profiles, and found that deletion of the genes most highly expressed in the hemispheres or vermis showed opposing effects on GCP differentiation.

Conclusion: We found that GCPs respond differentially to two driver mutations and a subset of GCPs is more susceptible to high level of SHH signaling as well as tumors formation. We redefined the main cell of origin by showing that GCPs are heterogeneous with molecularly distinct populations based on their location.

**PP49**

**The developmental sensitivity of vermis cerebelli to autistic chemical inducers; Rodent ASD model animal can help us or not?**

Yoshida S. ^1^, Iwamoto S. 1, Matsufusa R. ^1^, Fueta Y. ^2^, Ueno S. ^2^, Sekino Y. ^3^, Nomura Y. ^4^, Kanda Y. ^5^

^1^ Toyohashi University of Technology, Department of Environmental and Life Sciences, Toyohashi, Japan^2^ University of Occupational and Environmental Health, Fukuoka, Japan^3^ The University of Tokyo, Tokyo, Japan^4^ Queens College, City University of New York, New York, USA

^5^ National Institute of Health Sciences, Tokyo, Japan

Corresponding author’s e-mail: syoshida@ens.tut.ac.jp

Autism spectrum disorder (ASD), which is a severe neurodevelopmental disorder, is reported to show cerebral and cerebellar abnormalities. Multiple environmental agents, for example, sodium valproate (VPA) and chlorpyrifos (CPF), have been associated with an increased risk of ASD. In ASD, some structural abnormalities in the cerebellum, especially, a reduction in size and number of Purkinje cells is revealed in the human postmortem studies.

In this study, we established ASD-model rat with the administration to embryonic day 16 p.o. (VPA; 600mg/kg and CPF; 4.3 mg/kg of mother weight, respectively), and observed their cerebellar development and behavioral alteration. An animal placed on the open-field circle (80 cm in diameter) with an overhead camera, was observed 10 min. All tracking data was analyzed with Tracker Video Analysis and Modeling Tool produced by D. Brown.

VPA- or CPF-treated ASD animal models showed the excess development of Purkinje cells and excess folds in some cerebellar lobule were observed in 2 weeks after birth. This alteration was also observed in the VPA-administrated mature cerebellum. VPA-administrated or vehicle male rats from 6 to 8 weeks were tested their behavior, social interaction to same- or bigger-size strange rats and target interaction to well-known or new objects. In the individual behavior, VPA animals moved faster and longer than control. In the social interaction of bigger-size stranger, VPA animals showed fear than control and sometimes froze. In target behavior to a new object, VPA animals kept away. The grooming time of VPA animals was shorter than control. Consequently, VPA-administrated animals showed trends of hyperactive behavior in a reassured condition but felt fear to a strange situation, which behavior is similar to ADHD, not ASD. We suggest the ASD-induced animal models would help us understand the primary events of ASD, but might have some limitation of human ASD modification.

**PP50**

**Far-infrared radiation improves motor dysfunction and neuropathology in spinocerebellar ataxia type 3 mice**

Liu C.S. ^1,3,6*+^, Liu S.W. ^1+^, Chang J.C^. 1+^, Chuang S.F. ^1^, Liu K.H. ^1^, Cheng W.L. ^1^, Chang H.S. ^1^, Lin T.T. ^1^, Hsieh C.L. ^4,6^, Lin W.Y. ^5,6^, Hsieh M.L. ^7^, Kuo S.J. ^2^

^1^ Vascular and Genomic Center^2^ Department of Surgery^3^ Department of Neurology, Changhua Christian Hospital, Changhua 50094, Taiwan

^4^ Department of Chinese Medicine^5^ Departments of Medical Research, Obstetrics and Gynecology, Dermatology, and Urology, China Medical University Hospital, Taichung 40447, Taiwan^6^ School of Chinese Medicine, Graduate Institute of Chinese Medicine, Graduate Institute of Integrated Medicine, College of Chinese Medicine, Research Center for Chinese Medicine and Acupuncture, China Medical University, Taichung 40447, Taiwan^7^ Department of Life Science, Tunghai University, Taichung 40704, Taiwan

Corresponding author’s e-mail: 26602@cch.org.tw

Spinocerebellar ataxia type 3 (SCA3) is a polyglutamine neurodegenerative disease resulting from the misfolding and accumulation of a pathogenic protein, causing cerebellar dysfunction; and this disease currently has no effective treatments. Far-infrared radiation (FIR) has been found to protect the viability of SCA3 cells by preventing mutant ataxin-3 protein aggregation and promoting autophagy. However, this possible treatment still lacks in vivo evidence. This study assessed the effect of FIR therapy on SCA3 in vivo by using a mouse model over 28 weeks. Control mice carried a healthy wild-type ATXN3 allele that had a polyglutamine tract with 15 CAG repeats (15Q), whereas SCA3 transgenic mice possessed an allele with a pathological polyglutamine tract with expanded 84 CAG (84Q) repeats. The results showed that the 84Q SCA3 mice displayed impaired motor coordination, balance abilities, and gait performance, along with the associated loss of Purkinje cells in the cerebellum, compared with the normal 15Q controls; nevertheless, FIR treatment was sufficient to prevent those defects. FIR significantly improved performance in terms of maximal contact area, stride length, and base support in the forepaws, hindpaws, or both. Moreover, FIR treatment supported the survival of Purkinje cells in the cerebellum and promoted the autophagy, as reflected by the induction of autophagic markers, LC3II and Beclin-1, concomitant with the reduction of p62 and ataxin-3 accumulation in cerebellar Purkinje cells, which might partially contribute to the rescue mechanism. In summary, our results reveal that FIR confers therapeutic effects in an SCA3 transgenic animal model and therefore has considerable potential for future clinical use.

**OP1**

**The basal ganglia and the cerebellum: nodes in an integrated network.**

Bostan A.C. ^1^, Strick P.L.^ 1, 2^

^1^ Systems Neuroscience Center and Center for the Neural Basis of Cognition, University of Pittsburgh^2^ University of Pittsburgh Brain Institute and Departments of Neurobiology, Neuroscience and Psychiatry, University of Pittsburgh

Corresponding author’s e-mail: strickp@pitt.edu

The basal ganglia and the cerebellum are considered to be distinct subcortical systems that perform unique functional operations. The outputs of the basal ganglia and the cerebellum influence many of the same cortical areas, but do so by projecting to distinct thalamic nuclei. As a consequence, the two subcortical systems were thought to be independent and communicate only at the level of the cerebral cortex. We will review recent data showing that the basal ganglia and the cerebellum are interconnected at the subcortical level. The subthalamic nucleus in the basal ganglia is the source of a dense di-synaptic projection to the cerebellar cortex. Similarly, the dentate nucleus in the cerebellum is the source of a dense di-synaptic projection to the striatum. These observations lead to a new functional perspective that the basal ganglia, the cerebellum, and the cerebral cortex form an integrated network. This network is topographically organized so that the motor, cognitive, and affective territories of each node in the network are interconnected. This perspective explains how synaptic modifications or abnormal activity at one node can have network-wide effects. A future challenge is to define how the unique learning mechanisms at each network node interact to improve performance in the motor, cognitive and affective domains.

**OP2**

**Encoding of voluntary whisker movement at input and output stages of the cerebellar cortex**

Chadderton, Paul ^1^

^1^Department of Bioengineering, Imperial College London, London, UK

Corresponding author’s e-mail: p.chadderton@imperial.ac.uk

The cerebellum or ‘little brain’ is a major site of sensorimotor integration and contains more than half of all the neurons in the mammalian brain. The elegant repeating circuitry of the cerebellar cortex has led to its description as ‘a neuronal machine’, but we know surprising little about the circuit encodes sensory and motor information during behaviour. We have approached this problem by using a well-defined model, the mouse vibrissae system, to study how sensorimotor signals are represented by the activity of individual neurons within the cerebellar cortex. In this talk I will describe how whisking behaviour is encoded by neurons in both input and output layers of the cerebellar cortex. Patch clamp recordings from cerebellar granule cells (GCs) in the input layer reveal that movement is accompanied by changes in mossy fibre activity that drive membrane potential depolarisation and high-frequency bursting activity at preferred whisker angles. Although individual GCs are narrowly tuned, GC populations provide linear excitatory drive across a wide range of movement, ensuring that molecular layer interneurons and Purkinje cells exhibit bidirectional firing rate changes during whisking. Together, GC populations provide downstream PCs with linear representations of volitional movement, while inhibitory networks invert these signals. The exquisite sensitivity of neurons at each processing stage enables faithful propagation of kinematic representations through the cerebellum. Our results reveal that cells in the cerebellum use a simple linear code to represent voluntary whisker movements, providing a powerful new platform to explore neural computations underlying sensorimotor behavior.

**OP3**

**Cerebellar inhibition, prism adaptation, eye-hand coordination in ataxia**

Ugawa Y. ^1^

^1^Department of Neurology, Fukushima Medical University, Fukushima, Japan

Corresponding author’s e-mail: ugawa-tky@umin.net

In this communication, I would like to present three electrophysiological methods to study cerebellar function and dysfunction in humans.

Motor cortical inhibition by cerebellar stimulation (CBI): Magnetic stimulation over the cerebellum reduces the size of MEPs to motor cortical stimulation when given 5-8 ms prior to the motor cortical stimulation (CBI). CBI is shown to be produced by Purkinje cell activation by cerebellar stimulation through cerebello-thalamo-motor cortical pathways. The degree of abnormality of CBI positively correlated with ICARS score. The CBI must be used as one objective marker of classical cerebellar symptoms.

Prism adaptation: When visual inputs are angled by prism glasses, we are able to adapt motor tasks to a new angled visual situation by gradual learning. If the learning is incomplete, the residual error remains (asymptomatic error). When the grass was off after the adaptation completion, the movement was oppositely directed (the aftereffect). The asymptomatic error was significantly larger, and the aftereffect was significantly smaller in ataxic patients than normal subjects. The degree of abnormality did not positively correlate with the ICARS score. The prism adaptation may reflect cerebellar function of adaptation having no correlation with clinical cerebellar symptoms.

Eye-hand coordination: We devised a system which can record the trajectory of hand and eye movements simultaneously, when subjects performed a visually guided reaching task. In most trials, the eyes preceded the finger toward the target, and were locked at the target location until the hand reached there. The interval between the onsets of the eyes and the hand movements was larger for ataxic patient than for normal subjects, and this delay did not correlate with the ICARS score. The delay may also reflect some cerebellar function not seen by clinical cerebellar symptoms.

The above three physiological methods contribute to cerebellar functional analysis.

**OP4**

**Disease-modifying therapies for polyglutamine-mediated spinocerebellar ataxias.**

Katsuno M. ^1^, Hashizume A. ^1^, Hijikata Y. ^1^, Yamada S. ^1^, Ito D. ^1^

^1^Department of Neurology, Nagoya University Graduate School of Medicine, Nagoya, Japan

Corresponding author’s e-mail: ka2no@med.nagoya-u.ac.jp

Polyglutamine diseases are hereditary neurodegenerative disorders caused by an abnormal expansion of a trinucleotide CAG repeat, which encodes a polyglutamine tract. To date, nine polyglutamine diseases are identified: Huntington’s disease (HD), spinal and bulbar muscular atrophy (SBMA), dentatorubral-pallidoluysian atrophy, and six forms of spinocerebellar ataxia (SCA). We explored molecular signaling alteration underlying polyglutamine diseases, and found that Akt pathway is commonly involved in both SCA1 and SBMA, suggesting that up-regulation of Akt is a potential therapeutic approach for treating polyglutamine diseases.

Recently, mesenchymal stem cell (MSC) has been shown to ameliorate neurological symptom in mouse models of polyglutamine-SCAs, and clinical efficacy of this therapy is also shown in a phase1/2 trial targeting patients with Machado-Joseph disease. Cell therapy thus is a candidate of therapy for recovering motor function in polyglutamine-mediated SCAs. The precise mechanism of neuroprotection by MSC remains elusive, but they are shown to have potential to preferentially fuse with degenerating Purkinje cells in a mouse model of SCA1.

Lack of sensitive outcome measure is another critical component in clinical trials of disease-modifying therapies for SCAs, as the progression of these diseases is slow, and expected therapeutic effect over natural history is hard to be detected using conventional motor scores. We are developing a device to quantitatively evaluate upper limb ataxia using Geomagic Touch. This tool records time and position of a pen held by patients, enabling measurement of 3D movement of upper limbs. By adopting a task to press two buttons by the pen, we evaluated the length of trajectory and time, and found that both measures are elongated in patients with SCAs compared with healthy controls, suggesting that this device quantifies upper limb ataxia in clinical studies.

**OP5**

**Climbing fiber bursts encode stimulus features and are dynamically modulated during motor learning**

Fanning A. ^1^, Siegel J. ^1^, Chitwood R. ^1^, Johnston D. ^1^, Nishiyama H. ^1^

^1^ Center for Learning and Memory, Department of Neuroscience, The University of Texas at Austin, USA

Corresponding author’s e-mail: hiroshi@utexas.edu

Climbing fibers (CFs) are the axons originating from excitatory neurons in the inferior olive that provide instructive signals driving cerebellar-dependent motor learning. CFs are activated by erroneous movement or unexpected sensory stimuli providing error feedback to Purkinje cells (PCs) in cerebellar cortex and triggering plasticity that makes way for correcting subsequent movements. CFs exhibit variable, high-frequency bursts of 1-6 action potentials, yet the consequence of this variable activity has been largely ignored. This is because even a single action potential produces a massive all-or-none postsynaptic PC response. Recent evidence suggests that CF burst size may encode parametric features of a sensory stimulus and possibly determine the speed and direction of learning. However, in previous studies, CF burst size has been inferred based on PC recordings, which may be affected by factors other than CF activity, such as non-CF inputs to the PCs and the intrinsic excitability of the PCs. Using calcium (Ca2+) imaging to directly measure CF activity in awake mice, we show that CF burst size serves multiple functions. CFs differentiate unexpected sensory events from spontaneous activity by increasing burst size. Furthermore, CF burst sizes increases as the strength of sensory stimuli increases. During eyeblink conditioning, CFs are more reliably responsive to the unconditioned stimulus (US; air puff) before learning occurs, but switch to become more reliably responsive to the conditioned stimulus (CS; a light) as learning progresses. As the switch occurs, the magnitude of CS-evoked Ca2+ transients increases, whereas repeatedly presenting only the CS (unpaired) results in a decrease in the magnitude of Ca2+ transients. These data suggest that CF burst size serves multiple functions: to encode unexpected sensory stimuli, provide positive error feedback, and to attach saliency to cues that reliably predict aversive stimuli presentation.

**OP6**

**Zfp423 / ZNF423 regulates Purkinje cell and cerebellar nuclei development**

Consalez G.G. ^1,2^, Croci L. 2, Gaudesi D ^2^, Cremona O ^1,2^, Hawkes R. ^3^, Warming S. ^4^, Casoni F. ^1,2^

^1^ Division of Neuroscience, San Raffaele Scientific Institute, Milan, Italy^2^ Universita’ Vita-Salute San Raffaele, Milan, Italy

^3^ University of Calgary, Calgary, AB, Canada^4^ National Cancer Institute, Frederick, MD, USA

Corresponding author’s e-mail: g.consalez@gmail.com

The Zfp423/ZNF423 gene encodes a 30-zinc-finger transcription factor involved in key developmental pathways. Although null Zfp423 mutants develop cerebellar malformations, the underlying mechanism is only partially characterized. In humans, ZNF423 mutations are associated with cerebellar vermis hypoplasia and Joubert Syndrome (JS), a ciliopathy causing congenital ataxia. ZNF423 participates in the DNA-damage response (DDR), suggesting that its mutation may slow down neural progenitor cell cycle progression in cerebellar development. To characterize in vivo the function of ZFP423 in neurogenesis, we analysed allelic murine mutants in which distinct functional domains are deleted. In Purkinje cell (PC) progenitors, located in the cerebellar ventricular zone (VZ), the two mutations produce different alterations in mitotic spindle orientation, maintenance of the progenitor pool and neuronal differentiation. In both mutants, cell cycle progression is remarkably delayed and DDR markers are upregulated in VZ and rhombic lip (RL) progenitors. In the RL, Zfp423 mutants display an increase in cell death at key developmental stages, and clear alterations in cerebellar nuclei (CN) development. Our results reveal protein-domain-specific roles played by ZFP423 in different aspects of PC and CN neurogenesis, and at the same time strengthen the emerging notion that an impaired DDR may be a key factor in the pathogenesis of JS and other ciliopathies.

**OP7**

**Cerebellar Purkinje cells control movement with a rapid rate code that is invariant to spike regularity**

Hannah L. Payne, Ranran L. French, Christine C. Guo, T.D. Barbara Nguyen-Vu, Jennifer L. Raymond

Corresponding author’s e-mail: jenr@stanford.edu

Neurons transmit information through sequences of spikes, which are characterized by both mean firing rate and precise temporal pattern. Whereas firing rate is known to encode information and impact the activity of downstream neurons, the functional significance of spike pattern is less well understood. We used recording, stimulation, and computer modelling approaches to analyze how the fine temporal pattern of spikes in the oculomotor cerebellum affects motor output. Previous experimental and modelling studies have suggested that the regularity of interspike intervals in cerebellar Purkinje cells affects the transmission of information to downstream neurons and motor be-havior. During oculomotor behavior, recordings from awake behaving animals revealed that the regularity of Purkinje cell spiking, like the spike rate, was correlated with eye velocity in both mice and monkeys, suggesting that it might impact the control of eye movements. However, dissociation of the effects of spike rate and regularity through a residual analysis revealed no effect of spike regularity on motor output beyond the impact of spike rate alone. Further, when spike regularity was causally dissociated from spike rate using optogenetic stimulation, regular and irregular spike patterns with the same mean rate were equally effective at driving eye movements. A biophysical model of the synapse between Purkinje cells and their postsynaptic targets delineated the range of biologically realistic parameters for which Purkinje cell spike pattern impacts downstream neural activity. This work extends our understanding of the neural code by delineating the conditions un-der which neural spikes can reliably convey continuous information through rapid changes in rate, regardless of their exact temporal pattern.

**OP8**

**From distribution to localization of brain structural networks in different diseases stage of multiple system atrophy of the cerebellar type (MSA-C)**

Po-Shan Wang ^1,2^, Bing-Wen Soong ^3,4^ , Hsiu-Mei Wu ^5^, Chii-Wen Jao ^2*^, Yu-Te Wu ^2*^

^1^The Neurological Institute, Taipei Municipal Gan-Dau Hospital, Taipei, Taiwan, ROC^2^National Yang-Ming University, Taipei, Taiwan, ROC^3^The Neurological Institute, Taipei Veterans General Hospital, Taipei, Taiwan, ROC^4^Department of Neurology, National Yang-Ming University School of Medicine, Taipei, Taiwan, ROC^5^Department of Radiology, Taipei Veterans General Hospital, Taipei, Taiwan, ROC

Corresponding author’s e-mail: ytwu@ym.edu.tw

Background: Apart from cerebellar atrophy, patients with multiple system atrophy of the cerebellar type (MSA-C) also reveal disruption of cerebral atrophy and cognitive declines in verbal memory and executive function. These dysfunctions are usually associated with damage to the prefrontal cortex. Recently, network model has been proposed as a useful tool for investigating the structural organization and functional mechanisms for understanding clinical brain disorders. Till now, the brain structural network of patients with MSA-C is less explored. In this study, we aim to investigate the structural network in MSAC of early and late disease stages.

Method: Forty-two healthy subjects (42 ± 5 years, 22 male, 20 female) and forty-nine MSA-C patients (51.8 ± 7 years, 25 male, 24 female) participated in this study. The mean illness duration was 4.3 ± 2.8 years. We subdivided the patients into two groups: early MSA-C group (9 patients, disease duration less than 3 years) and the late MSA-C group (40 patients, disease duration longer than 3 years). Each voxel of cerebral and cerebellar gray matter was anatomically aligned to the 97 anatomical label (AAL) structures by using IBASPM (Left cerebrum: 45, right cerebrum: 45, cerebellum: 7). Subsequently, the 3-dimension fractal dimension (3D-FD) values of parcellated 97 regions and modular analysis were employed to categorize and construct the structural network. Small-world properties were compared via permutation test to evaluate the significant difference between groups.

Results: The early MSAC group showed highly significant atrophy in the whole cerebellum and basal ganglia (putamen and pallidum) (p < 0.001), and significant atrophy in some regions of frontal, occipital and parietal lobes (p < 0.05).The late MSA-C group showed more atrophied regions in the same atrophied lobes of early MSA-C, and revealed more atrophied regions in limbic, occipital and temporal lobes. The number of clustered modules of network increased with disease duration (normal: 5, early MSA-C: 6 late MSA-C: 7), and the modularity (Q) of network decreased with disease duration (normal: 0.2956, early MSA-C: 0.2827, late MSA-C: 0.2426, p < 0.05). In normal, the sub-regions of cerebellum were clustered into three different major functional modules. There is at least one connector hub in each module that connects with other modules. The normal group recruited more different functional lobes in the major functional modules and exhibited dense linkages in commutation with each other module. Different from the normal group, both early and late MSA-C groups recruited fewer lobes in their major functional modules as compared with normal group. The early and late MSA-C groups revealed more overlapped regions in the three functional modules. Only three modules of their networks have connector hubs (modules with connector hub/ total modules: early MSAC: 3/6, late MSAC: 3/7). In early MSA-C, the whole cerebellum, superior temporal gyrus and angular gyrus were clustered into an isolated module (no connector hub within the module). As the disease duration increasing, the superior temporal gyrus and angular gyrus separated from the module and the cerebellum of late MSAC was isolated to form a single module. Both early and late MSAC groups revealed the tendency of grouping neighbored lobes to form modules. In other words, these modules consist of more local regions instead of distinct ones. Such a module formed by local regions may result in shortening the practical distance between nodes within module. However, we found that both the early and late MSA-C groups manifest longer characteristic path length than the normal group.

Conclusions: The modules formed by local regions may imply the transaction of brain network from functional connectivity to anatomical connection in different stages of MSA-C. The decreasing of modularity and increasing of characteristic path length provides an explanation to the continuous loss of efficiency of network at different stages of MSA-C. The distributed atrophy in cerebrum and segregated regions in major functional modules between normal group and early MSA-C may cause the cognitive dysfunction of MSA-C.

**OP9**

**Cerebellar dizziness and cerebellar ocular motor disorders: diagnosis and current pharmacotherapy**

Strupp Michael ^1^, Feil Katharina ^1^, Bremova Tatiana ^1^

^1^Department of Neurology, University Hospital, Munich and German Center for Vertigo and Balance Disorders, Hospital of the LMU Munich, Germany

Corresponding author’s e-mail: michael.strupp@med.uni-muenchen.de

Objectives. To identify patients with vertigo caused by different cerebellar diseases.

Methods. The medical records of all patients diagnosed with cerebellar disease in the German Center for Vertigo between 2011 and 2015 were reviewed.

Results. 463 patients (215 men) out of 5400 patients presenting with cerebellar dizziness were included (mean age 65.5 ± 15.6 yrs). 84.1% reported having permanent dizziness, 31.8% reported having attacks of vertigo and 16.2% reported having both. 46.2% of patients reported a subjective progression of the symptoms over time. 66.5% of patients presented with unsteadiness of gait, 19.4% (n = 90). Ocular motor disturbances like diplopia, oscillopsia and blurred vision were reported in 37.8% (n = 175) and speech disturbances in 19.4% (n = 90). 35.9% of patients had isolated mild to moderate cerebellar ocular motor disturbances without any limb ataxia, dysarthria or other sign of cerebellar ataxia followed by downbeat nystagmus syndrome in 24.2% and cerebellar ataxia in 20.5%; CAVAS was diagnosed in 11.0% of cases. 87.1% of patients had saccadic smooth pursuit and 28.1% strabism (which can also be a cerebellar sign). Saccades, either hyper- or hypometric, were pathological in 42.3%. Finally, 45.1% had a pathological fixation suppression of the VOR. MRI examinations were available in 41% of cases (n = 190) and considered to be pathological in 106 cases (56 patients with cerebellar atrophy). Two hundred sixty-nine patients underwent posturography, with all of them having pathological results, with a 3-Hz sway typical of cerebellar vermal dysfunction in 15.6%.

Conclusions. Dysfunction of the cerebellum is a relatively frequent cause of vertigo and dizziness. One third of the 463 patients with cerebellar vertigo and dizziness had isolated cerebellar ocular motor dysfunction only, one fifth of the patients had downbeat nystagmus syndrome. Therefore, from a clinical point of view, a careful examination of eye movements and nystagmus is often the key to the diagnosis.

**OP10**

**Where's the receptor? - finding NMDA receptors responsible for motor learning and long-term depression in the cerebellum**

Kono M. ^1,2^, Kakegawa W. ^2^, Yuzaki M. ^2^

^1^Department of neurosurgery, Keio University School of Medicine, Tokyo, Japan^2^Department of Physiology, Keio University School of Medicine, Tokyo, Japan

Corresponding author’s e-mail: maya@keio.jp

NMDA receptors are ionotropic glutamate receptors expressed in various brain regions. It plays crucial roles in synaptic plasticity, including long-term potentiation (LTP) and depression (LTD), cellular models of learning and memory (Nicoll and Malenka, Ann N Y Acad Sci, 1999). In the cerebellum, NMDA receptors are also expressed in several types of neurons, such as granule cells (GCs), molecular layer interneurons (MLIs) and Purkinje cells (PCs). Previous studies using pharmacological tools and gene knockout (KO) mice demonstrated that NMDA receptors regulate cerebellar LTD and motor learning through a nitric oxide/protein kinase G pathway. Nevertheless, it has remained unclear where and how NMDA receptors mediate these functions. To resolve this long-standing controversy, we generated three kinds of cell-specific conditional KO mice of GluN1, an obligatory subunit of NMDA receptors, by crossing GluN1-flox mice with various cell-specific Cre-driver mice. GC-specific GluN1 KO mice exhibited a robust LTD as well as LTP at parallel fiber-PC synapses. Similarly, PC-specific GluN1 KO mice showed normal cerebellar LTD/LTP. These mice also showed normal adaptation of horizontal optokinetic responses (hOKR), a representative cerebellar motor learning task. In contrast, mice in which GluN1 was knocked out in parvalbumin-positive MLIs and PCs displayed severely impaired LTD and hOKR adaptation, while these mice showed grossly normal cerebellar anatomy, motor coordination and cerebellar LTP. These results indicate that it is the NMDA receptor expressed in MLIs, but not in PCs or GCs, that is required for cerebellar LTD and motor learning in vivo.

**OP11**

**Cerebral Consequences of Cerebellar Degeneration: Functional and Structural MRI Studies in Friedreich Ataxia**

Harding, IH ^1^

^1^Monash Institute of Cognitive & Clinical Neurosciences, Monash University, Melbourne, Australia

Corresponding author’s e-mail: ian.harding@monash.edu

Friedreich ataxia (FRDA) is the most common inherited ataxia, characterised by progressive motor incoordination secondary to degeneration of the dentate nuclei of the cerebellum and dorsal spinal tracts. The cerebrum has traditionally been thought to be spared in FRDA, with little evidence of cortical grey matter pathology. However, the dentate nuclei are the primary source of long-range cerebello-cerebral projections in humans, giving rise to dentato-thalamo-cerebral white matter pathways and extensively innervating the cerebral cortex. In this talk, I will present a series of functional and diffusion MRI studies that examine cerebro-cerebellar function and connectivity in individuals with FRDA. The results of this work challenge traditional conceptions of isolated cerebellar deficits in FRDA, but indicate that the downstream functional consequences of cerebellar atrophy are not universally detrimental, and may also reflect compensatory processes in some contexts.

**OP12**

**Molecular/cellular mechanisms underlying activity-dependent climbing fiber synapse elimination in the developing cerebellum**

Masanobu Kano^1^

^1^Department of Neurophysiology, Graduate School of Medicine, The University of Tokyo

Corresponding author’s e-mail: mkano-tky@m.u-tokyo.ac.jp

Purkinje cells (PCs) in the neonatal cerebellum of rodent receive excitatory synaptic inputs from multiple climbing fibers (CFs). During the first three postnatal weeks, redundant CFs are eliminated and most PCs become innervated by single CFs. This developmental synapse elimination consists of at least four distinct phases: (1) selective strengthening of a single CF out of multiple CFs innervating each PC from postnatal day 3 (P3) to around P7 (functional differentiation), (2) expansion of innervation territory of the strengthened CF (‘winner’ CF) from P9 (dendritic translocation), (3) elimination of somatic synapses of the ‘winner’ CF and of weaker CFs (‘loser’ CFs) from P7 to around P11 (early phase of CF elimination), (4) elimination of somatic synapses of the ‘winner’ CF and of ‘loser’ CFs from around P12 to P17 (late phase of CF elimination). We have been investigating molecular/cellular mechanisms underlying these distinct phases of CF synapse elimination. We have shown that P/Q-type voltage-dependent calcium channel (VDCC), the major VDCC in PCs, is essential for the functional differentiation, the early phase of CF elimination and the dendritic translocation of a single ‘winner’ CF. We have also demonstrated that the late phase of CF elimination requires the type 1 metabotropic glutamate receptor (mGluR1) to protein kinase C_gamma cascade in PCs, involves activation of the immediate early gene Arc, and is regulated by GABAergic inhibition of the PC soma by basket cells. These results indicate that CF synapse elimination is critically dependent on the activity of PCs, and suggest the presence of retrograde signaling mechanisms from postsynaptic PCs to presynaptic CF terminals. I will present new results from our on-going research and discuss how CF mono innervation in the mature cerebellum is established through multiple activity-dependent processes which may involve distinct retrograde signaling mechanisms.

**OP13**

**Cerebello-cerebral interactions for planning and choosing motor programs.**

Chris De Zeeuw^**1**^

^1^ Erasmus University Rotterdam, Department of Neurocience

Corresponding author’s e-mail: c.dezeeuw@erasmusmc.nl

The brain can store information in persistent neural activity to remember past events and plan future behavior. Persistent and ramping activity in frontal cortex reflects the anticipation of specific movements. This preparatory activity has long been postulated to emerge from processes distributed across multiple brain regions, but it has remained largely unclear how this activity is mediated by multi-regional interactions and which brain areas are involved. For this lecture, I will describe how a persistent representation of information in the frontal cortex critically depends on cerebellar processing, a brain structure originally thought to be primarily involved in online control of movement. During a sensory discrimination task, in which mice use short-term memory to plan a future directional movement of their tongue, they show persistent ramping activity in both their frontal cortex and cerebellar nuclei, instructing future movements seconds before their onset. Transient perturbations in activity of the medial cerebellar nucleus disrupt these ramping activities as well as their choices to move their tongue in the right direction. Moreover, silencing frontal cortex activity abolishes preparatory activity in the cerebellar nuclei affecting a closed cortico-cerebellar loop. Finally, ongoing motor programs in this closed loop can be altered by manipulating activity in the olivocerebellar system, resetting the planned behavior. Together, these experiments highlight the way the cerebellum can control cognitive behaviors that extend beyond coordination of ongoing movements.

